# STAT3 drives the expression of ACSL4 in acute kidney injury

**DOI:** 10.1016/j.isci.2024.109737

**Published:** 2024-04-16

**Authors:** Virginie Poindessous, Helene Lazareth, Gilles Crambert, Lydie Cheval, Julio L. Sampaio, Nicolas Pallet

**Affiliations:** 1 Centre de Recherche des Cordeliers, INSERM U1138, Université Paris Cité, Paris, France; 2 Université Paris-Cité, Paris, France; 3 Laboratory of Renal Physiology and Tubulopathies, Centre de Recherche des Cordeliers, INSERM U1138, Sorbonne Université, Université Paris Cité, Paris, France; 4 EMR 8228 Metabolism and Renal Physiology Unit, CNRS, Paris, France; 5 CurieCoreTech Metabolomics and Lipidomics Technology Platform, Institut Curie, Paris, France; 6 Department of Clinical Chemistry, Assistance Publique Hôpitaux de Paris, Georges Pompidou European Hospital, Paris, France

**Keywords:** pathophysiology, Integrative aspects of cell biology, omics

## Abstract

Long-chain acyl-CoA synthetase family 4 (ACSL4) metabolizes long-chain polyunsaturated fatty acids (PUFAs), enriching cell membranes with phospholipids susceptible to peroxidation and drive ferroptosis. The role of ACSL4 and ferroptosis upon endoplasmic-reticulum (ER)-stress-induced acute kidney injury (AKI) is unknown. We used lipidomic, molecular, and cellular biology approaches along with a mouse model of AKI induced by ER stress to investigate the role of ACSL4 regulation in membrane lipidome remodeling in the injured tubular epithelium. Tubular epithelial cells (TECs) activate ACSL4 in response to STAT3 signaling. In this context, TEC membrane lipidome is remodeled toward PUFA-enriched triglycerides instead of PUFA-bearing phospholipids. TECs expressing ACSL4 in this setting are not vulnerable to ferroptosis. Thus, ACSL4 activity in TECs is driven by STAT3 signaling, but ACSL4 alone is not enough to sensitize ferroptosis, highlighting the significance of the biological context associated with the study model.

## Highlights

• ACSL4 expression is induced in ER-stressed kidney tubular cells *in vivo* but not *in vitro*
• ACSL4 is upregulated in response to STAT3 signaling
• In this context, TECs lipidome is remodeled toward PUFA-enriched triglycerides
• TECs expressing ACSL4 in this setting are not vulnerable to ferroptosis

## Graphical abstract

**Figure:**
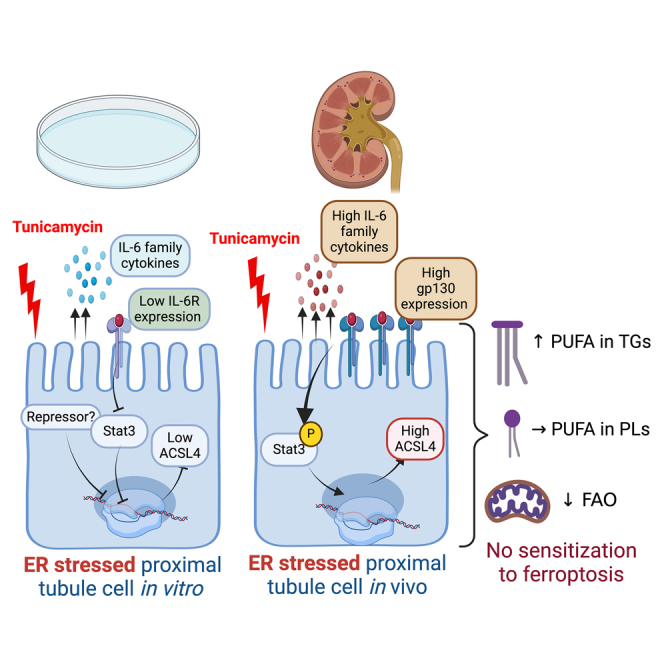

## Introduction

Impaired lipid metabolism in the proximal tubule is a fundamental aspect of acute kidney injury (AKI) and drives chronic epithelial kidney injury, leading to chronic kidney disease (CKD).^1^^,^^2^^,^^3^ Alterations in lipid metabolism in kidney tubular epithelial cells (TECs) occur as a consequence of extrinsic factors that promote kidney injury, such as ischemia/reperfusion (I/R), but also mediate cellular dysfunction (impaired cellular signaling, induction of an inflammatory/fibrogenic phenotype, and activation of regulated cell death) through multiple pathways, including increased fatty acids (FA) availability.^4^^,^^5^^,^^6^^,^^7^ FAs are stored as triacylglycerols (TAGs) in cytoplasmic lipid droplets.^8^^,^^9^ However, lipolysis of intracellular TAG stores and the TAG synthesis pathway can produce FAs and lipid intermediates with deleterious properties, and although the formation of long-chain acyl-CoAs can reduce cellular FA levels, acyl-CoAs themselves are problematic because they can act as detergents and are precursors of damaging lipid species such as ceramides and diacylglycerols.^9^^,^^10^ Due to their low reactivity, FAs must be activated to fatty acyl-CoA esters by acyl-CoA synthases for further metabolism: FA oxidation (FAO), phospholipid production, or storage in TAGs. Members of the long-chain acyl-CoA synthetase (ACSL) family include five different ACSL isoforms located on the endoplasmic reticulum (ER), mitochondrial outer membrane, cytosol, or plasma membranes and catalyze FAs with chain lengths of 12–22 carbon atoms to form acyl-CoAs.^11^^,^^12^

ACSL4 activates long-chain polyunsaturated fatty acids (PUFAs) to fatty acyl-CoA products, preferentially using arachidonate (C20:4, AA) and docosatetraenoate (C22:4, adrenic acid, AdA) as substrates.^13^ ACSL4 enriches cell membranes with AA- and AdA-containing phosphatidylethanolamine (PE) species that can be oxidized and accelerate cell death.^9^^,^^14^^,^^15^ Indeed, these phospholipid-bound PUFAs are known to be highly susceptible to peroxidation due to the presence of weak C-H bonds between adjacent C=C double bonds.^16^ Thus, ACSL4 is a critical determinant of susceptibility to ferroptosis, a regulated cell death pathway associated with the dysregulation of lipid oxidative metabolism that occur in the kidney and is implicated in the pathophysiology of acute and chronic kidney injury, especially in cases of I/R injury and diabetic nephropathy.^15^^,^^17^^,^^18^^,^^19^^,^^20^^,^^21^^,^^22^

Among the many factors involved in the early transcriptional response to kidney injury, and which profoundly alters lipid metabolism and induces cell death, the endoplasmic reticulum (ER) stress response plays a critical role, leading to tissue damage and loss of renal function.^23^^,^^24^ Impact of ER stress on ACSL4 activity and its regulatory mechanisms of ferroptosis in AKI are unknown.

## Results

### ER stress upregulates ACSL4 *in vivo* in tubular epithelial cells

**Figure 1:**
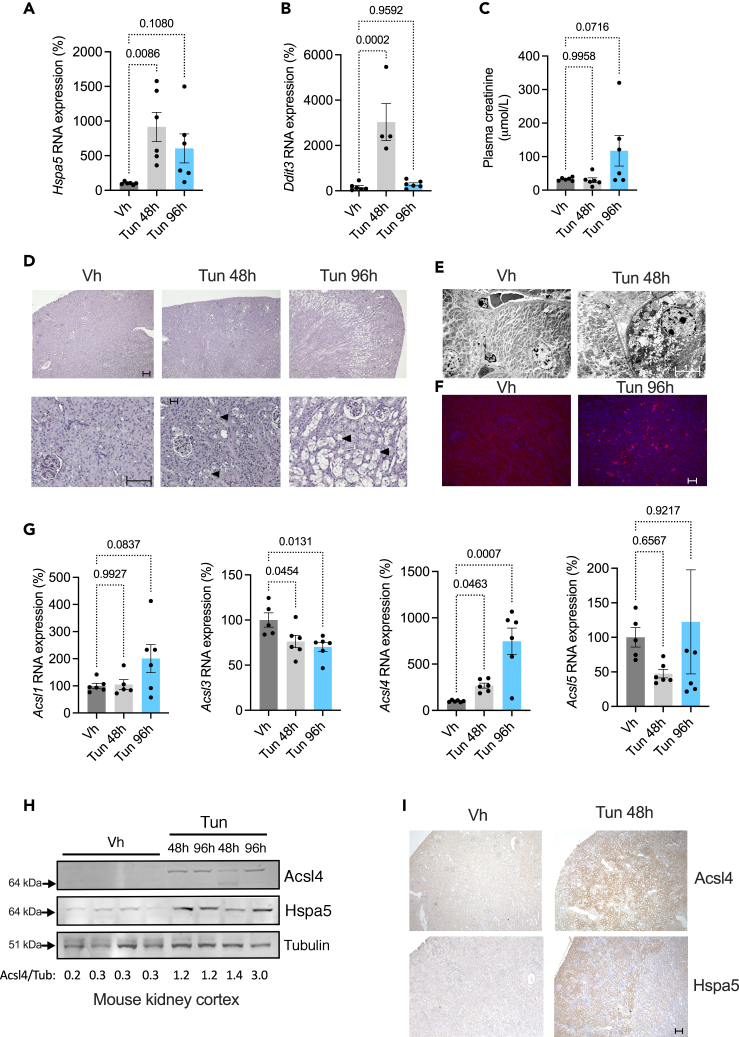
ER stress upregulates ACSL4 *in vivo* in tubular epithelial cells (A) *Hspa5* transcript levels measured by qPCR in kidney cortex of mice 48 and 96 h after intraperitoneal injection of 1 mg/kg tunicamycin (Tun) or DMSO (Vh) (n = 6 mice per group). p values were computed with one-way ANOVA followed by a Dunnett’s multiple comparisons test. Data are represented as mean ± SEM. (B) *Ddit3* transcripts levels measured by qPCR in kidney cortex of mice 48 and 96 h after intraperitoneal injection of 1 mg/kg tunicamycin (Tun) or DMSO (Vh) (n = 6 mice per group). p values were computed with one-way ANOVA followed by a Dunnett’s multiple comparisons test. Data are represented as mean ± SEM. (C) Plasma creatinine measurement (enzymatic method) in mice 48 and 96 h after intraperitoneal injection of 1 mg/kg tunicamycin (Tun) or DMSO (Vh) (n = 6 mice per group). p values were computed with one-way ANOVA followed by a Dunnett’s multiple comparisons test. Data are represented as mean ± SEM. (D) Periodic acid Schiff staining of kidney cortex of mice 48 h and 96 h after intraperitoneal injection of 1 mg/kg tunicamycin (Tun) or DMSO (Vh). Scale bar: 150 nm. Arrowheads denotes vacuolization. (E) Ultrastructure of kidney tubule cells by transmission electron microscopy from kidney cortex of mice 48 h after intraperitoneal injection of 1 mg/kg Tunicamycin (Tun) or DMSO (Vh). Scale bar: 5 μm. (F) DNA fragmentation visualization by TUNEL staining from kidney cortex of mice 96 h after intraperitoneal injection of 1 mg/kg Tunicamycin (Tun) or DMSO (Vh). Nuclei were counterstained with DAPI. Scale bar: 150 nm. (G) *Acsl1*, *Acsl3*, *Acsl4*, *and Acsl5* transcripts levels measured by qPCR in kidney cortex of mice 48 and 96 h after intraperitoneal injection of 1 mg/kg tunicamycin (Tun) or DMSO (Vh) (n = 6 mice per group). p values were computed with one-way ANOVA followed by a Dunnett’s multiple comparisons test. Data are represented as mean ± SEM. (H) Acsl4, Hspa5, and tubulin protein levels in kidney cortex of mice 48 h or 96 h after intraperitoneal injection of 1 mg/kg tunicamycin (Tun) or DMSO (Vh). The immunoblot is representative of three independent experiments. (I) Acsl4 and Hspa5 expression detected by immunohistochemistry in kidney cortex of mice 48 h after intraperitoneal injection of 1 mg/kg tunicamycin (Tun) or DMSO (Vh). Original magnification x4. Scale bar: 150 nm.

The ER is the major site of lipid synthesis in the cell, and ACSL4 is an ER-resident protein.^13^ We thus hypothesized that disruption of ER homeostasis might affect the activity of ACSL4. Mice that received a single injection of 1 mg/kg tunicamycin (Tun), an antibiotic that inhibits glycoprotein synthesis and activates the unfolded protein response (UPR), developed severe AKI associated with ER stress, with ER stress preceding kidney dysfunction (Figures 1A–1C). Macroscopic examination of kidney cortical sections stained with periodic acid-Schiff revealed the presence of macroscopic cytoplasmic vacuoles in TECs 48 h after Tun injection, which ended up occupying almost the entire cytoplasm at 96 h (Figure 1D). The most altered tubules were located in the highly sensitive corticomedullary junction. Ultrastructural examination of cortical epithelial cells by electron microscopy revealed that these vacuoles were lipid droplets (spherical organelles with a low electron density homogeneous lipid ester core and a surface phospholipid monolayer^25^^,^^26^) (Figure 1E). TUNEL staining revealed a significant level of DNA damage in epithelial cells in Tun-treated mouse kidneys, consistent with ongoing cell death process (Figure 1F).

In this injured environment, *Acsl4* transcripts, but not the other isoforms *Acsl1*, *3*, and *5* (*Acsl2* does not exist, and we were unable to design functional primers for *Acsl6*), and *Acsl4* proteins were increased in bulk kidney preparations and predominantly in the cortical tubule epithelium (Figures 1G–1I). The major ferroptotic genes *Fsp1* (Ferroptosis suppressor protein 1) and *Gpx4* (glutathione peroxidase 4), which reduce lipid peroxidation and ferroptosis, were not affected by ER stress, whereas the cystine transporter *Slc7A11* that maintains the cellular levels of reduced glutathione and suppress ferroptosis, was significantly upregulated (Figure S1). These results indicate that in a pure model of AKI associated with ER stress, *Acsl4* expression is induced at the transcriptional level in the epithelium, but without the other key regulators of ferroptosis.

### ER stress leads to the downregulation of *ACSL4 in vitro*

**Figure 2:**
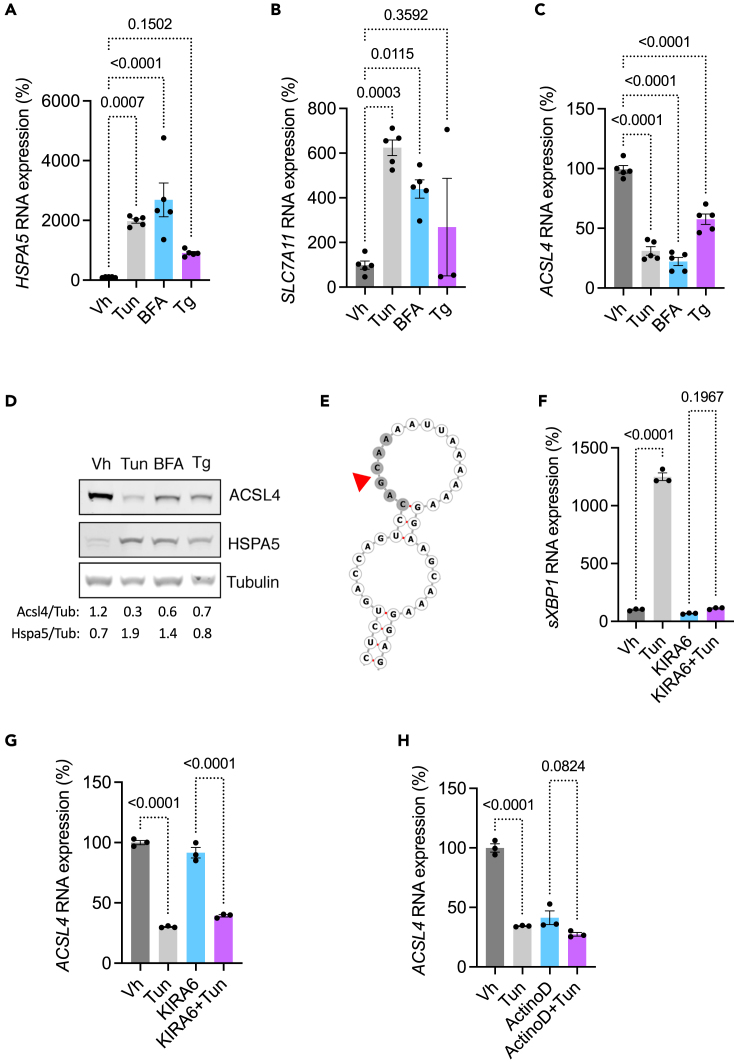
ER stress leads to the downregulation of *ACSL4in vitro* (A–C) *HSPA5*, *SLC7A11*, and *ACSL4* transcripts levels measured by RT-qPCR in HK-2 cells incubated with 2.5 μg/mL tunicamycin (Tun), 5 μg/mL brefeldin A (BFA), 0.25 μM thapsigargin (Tg), or DMSO (Vh) for 24 h (n = 5 replicates per condition). p values were calculated with a one-way ANOVA followed by a Dunnett’s multiple comparisons test. Data are represented as mean ± SEM. (D) ACSL4, HSPA5, and tubulin protein levels in HK-2 cells incubated with 2.5 μg/mL tunicamycin (Tun), 5 μg/mL brefeldin A (BFA), 0.25 μM thapsigargin (Tg), or DMSO (Vh) for 24 h (n = 5 replicates per condition). The immunoblot shown is representative of three independent experiments. (E) RNAfold prediction of secondary structure of mRNA fragments of *ACSL4* mRNA. Arrow head denotes the cleavage site. (F) *spliced XBP1 (sXBP1)* transcripts measured by RT-qPCR in HK-2 cells incubated with 2.5 μg/mL tunicamycin (Tun) or DMSO (Vh) for 24 h in the presence or absence of 1 μM KIRA6 (n = 3 replicates per condition). p values were computed with a one-way ANOVA followed by a Šídák multiple comparisons test. Data are represented as mean ± SEM. (G) *Acsl4* transcripts measured by RT-qPCR in HK-2 cells incubated with 2.5 μg/mL tunicamycin (Tun) or DMSO (Vh) for 24 h in the presence or absence of 1 μM KIRA6 (n = 3 replicates per condition). p values were computed with a one-way ANOVA followed by a Šídák multiple comparisons test. Data are represented as mean ± SEM. (H) Expression of *ACSL4* transcripts measured by RT-qPCR in HK-2 cells incubated with 2.5 μg/mL tunicamycin (Tun) or DMSO (Vh) for 24 h in the presence or absence of 0.05 μM actinomycin D (n = 3 replicates per condition). p values were computed with a one-way ANOVA followed by a Šídák multiple comparisons test. Data are represented as mean ± SEM.

To characterize the biological mechanisms underlying the induction of *ACSL4* expression in the cortical kidney epithelium upon ER stress, we performed *in vitro* studies in human kidney 2 (HK-2) cells of ER stress induced by Tun, brefeldin A (BFA, a lactone that inhibits protein transport from the ER to the Golgi complex), and thapsigargin (Tg, an inhibitor of sarco/endoplasmic reticulum Ca2+-ATPase) activated the UPR, but unexpectedly repressed *ACSL4* expression in HK-2 cells (Figures 2A–2D). The UPR may affect the expression of second messengers at the post-transcriptional level through IRE1α-mediated RNA decay (RIDD).^27^ *In silico* analysis of *ACSL4* mRNA secondary structures using the RNAfold web server (http://rna.tbi.univie.ac.at) predicted that it contains an IRE1α consensus sequence 5′-CAG↓CAA-3′ at position 1636-1644 of the coding sequence, located within a stem-loop secondary structure, suggesting that *ACSL4* mRNA may be an RIDD target (Figure 2E). We then tested the ability of IRE1α to cleave *ACSL4* mRNA by chemically inhibiting the RNase activity of IRE1α using KIRA6, an ATP-competitive IRE1α kinase inhibitor. KIRA6 did not prevent the downregulation of *ACSL4* mRNA, whereas it strongly inhibited the cleavage of *XBP1* to the spliced form *sXBP1*, indicating that *ACSL4* mRNA is not a *bona fide* RIDD target in HK-2 cells (Figures 2F and 2G). UPR signaling also involves mechanisms that attenuate or repress transcription through the ability of the proapoptotic transcription factor DDIT3 (CCAAT/enhancer binding protein [C/EBP] zeta, also known as CHOP) to form heterodimers with other C/EBP family members, resulting in inhibited transcription by titration of these transcription factors.^28^^,^^29^ Critically, like many genes involved in lipid metabolism, the 5′ region of the *ACSL4* gene is highly enriched for CEB/P consensus binding sites, suggesting that these transcription factors regulate *ACSL4* expression (Figure S2). To test whether the ER-stress-dependent decrease in *ACSL4* expression resulted from decreased gene transcription, we treated HK-2 cells with Tun in the presence or absence of the transcription inhibitor actinomycin D. Actinomycin D, like Tun, decreased *ACSL4* transcript levels in HK-2 cells to a similar extent (60%), but the addition of actinomycin D to Tun did not have a significant additive effect, suggesting that Tun affects *ACSL4* expression at the transcriptional level (Figure 2H). These results indicate that activation of the UPR in cultured TECs (HK-2 cells) represses *ACSL4* transcription in a cell-intrinsic manner.

### STAT3 signaling promotes the expression of ACSL4 in tubular epithelial cells

A possible explanation for the apparently contradictory effects of ER stress on the expression of *ACSL4* in cultured TECs and in TECs *in vivo* is the presence of extrinsic factors that are locally generated at high concentrations during the activation of the UPR, conditions that cannot be reproduced in the cell culture model. Among the molecules secreted by ER-stressed cells, the interleukin (IL)-6 cytokine family has been identified as being involved in the pathophysiology of kidney disease, particularly since these cytokines activate signal transducer and activator of transcription 3 (STAT3) signaling in the tubular epithelium, which drives tissue remodeling.^30^^,^^31^^,^^32^^,^^33^^,^^34^^,^^35^ Critically, the promoter region of *ACSL4* is enriched for STAT3 consensus binding sites (Figure S2).

**Figure 3:**
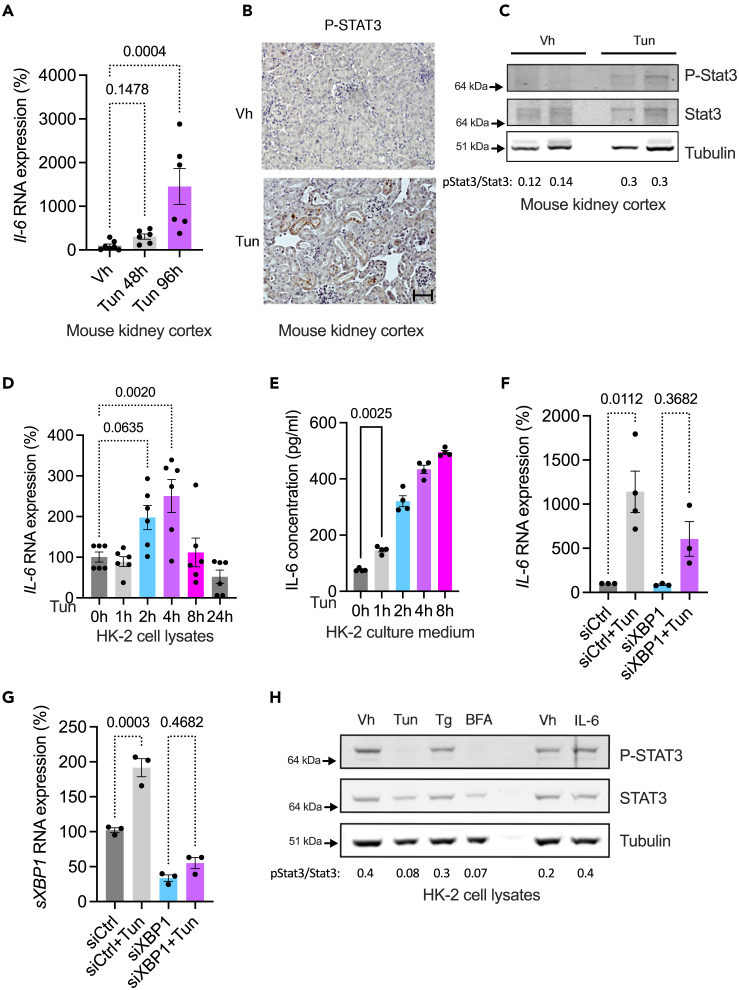
ER-stressed tubular epithelial cells produce IL-6 (A) Expression of *Il-6* transcripts by qPCR in kidney cortex of mice 48 h and 96 h after intraperitoneal injection of 1 mg/kg tunicamycin (Tun) or DMSO (Vh) (n = 6 mice per group). p values were computed with a one-way ANOVA followed by a Dunnett’s multiple comparisons test. Data are represented as mean ± SEM. (B) Phospho-STAT3 expression by immunohistochemistry in kidney cortex of mice 48 h after intraperitoneal injection of 1 mg/kg tunicamycin (Tun) or DMSO (Vh). Scale bar: 150 nm. (C) Phospho-STAT3 (P-STAT3), STAT3, and tubulin protein levels in kidney cortex of mice 48 h after intraperitoneal injection of 1 mg/kg tunicamycin (Tun) or DMSO (Vh). The immunoblot shown is representative of three independent experiments. (D) Time course analysis of the expression of *IL-6* transcripts measured by RT-qPCR in HK-2 cells incubated with 2.5 μg/mL tunicamycin (Tun) (n = 6 replicates per condition). p values were calculated with a one-way ANOVA followed by a Dunnett’s multiple comparisons test. Data are represented as mean ± SEM. (E) Time course analysis of the expression of concentration of IL-6 measured by ELISA in the culture medium of HK-2 cells incubated with 2.5 μg/mL tunicamycin (Tun) (n = 4 replicates per condition). p values were calculated with a one-way ANOVA followed by a Dunnett’s multiple comparisons test. Data are represented as mean ± SEM. (F) *IL-6* transcripts measured by RT-qPCR in HK-2 cells incubated with 2.5 μg/mL tunicamycin (Tun) or DMSO (Vh) for 24 h and transfected with siRNA directed against *sXBP1* RNA (siXBP1) or with scrambled siRNA (siCtrl) (n = 3 replicates per condition). p values were computed with a one-way ANOVA followed by a Šídák multiple comparisons test. Data are represented as mean ± SEM. (G) *sXBP1* transcripts measured by RT-qPCR in HK-2 cells incubated with 2.5 μg/mL tunicamycin (Tun) or DMSO (Vh) for 24 h (*n* = 3 replicates per condition) and transfected with siRNA directed against *sXBP1* RNA (siXBP1) or with scrambled siRNA (siCtrl). p values were computed with a one-way ANOVA followed by a Šídák multiple comparisons test. Data are represented as mean ± SEM. (H) Phospho-STAT3 (P-STAT3), STAT3, and tubulin protein levels in HK-2 cells incubated with 2.5 μg/mL tunicamycin (Tun), 5 μg/mL brefeldin A (BFA), 0.25 μM thapsigargin (Tg), 100 ng/mL IL-6 or vehicle (Vh) for 24 h. The immunoblot shown is representative of three independent experiments.

We observed that *Il-6* transcripts were produced in the kidneys of mice treated with Tun (Figure 3A) and that Stat3 was phosphorylated in the epithelium of injured kidneys, indicating that Stat3 signaling is active in ER-stressed tubules *in vivo* (Figures 3B and 3C). Since TECs release a variety of factors that cause autocrine signaling, we tested whether ER-stressed Tecs secrete IL-6. We incubated HK-2 cells with Tun and found that IL-6 was synthesized (Figure 3D) and secreted into the culture medium (Figure 3E) and that IL-6 production was partially sXBP1 dependent (Figures 3F and 3G), indicating that the UPR drives IL-6 production in TECs in culture. However, STAT3 was not phosphorylated in HK-2 cells incubated with various ER stressors, although these cells are responsive to IL-6 (Figure 3H). This observation can be explained by the fact that the concentrations of IL-6 measured in the culture medium of ER-stressed TECs are a thousand times lower than those used to induce an activation, albeit moderate, of STAT3 (Figures 3E and 3H). In addition, this apparent discrepancy may be linked to the regulation of IL-6 receptors expression. For example, HK-2 cells incubated with stress inducers fail to induce the express of IL6ST (GP130, a signal transducer needed for IL-6 receptor activity and signal transduction of the IL-6-related cytokines) and IL-6R (Figures S3A and S3B). Conversely, IL6ST is very strongly overexpressed in kidney cortex from mice exposed to tunicamycin (Figures S3D and S3E), which may underlie a difference in tissue sensitivity to IL-6-dependent signaling.

**Figure 4:**
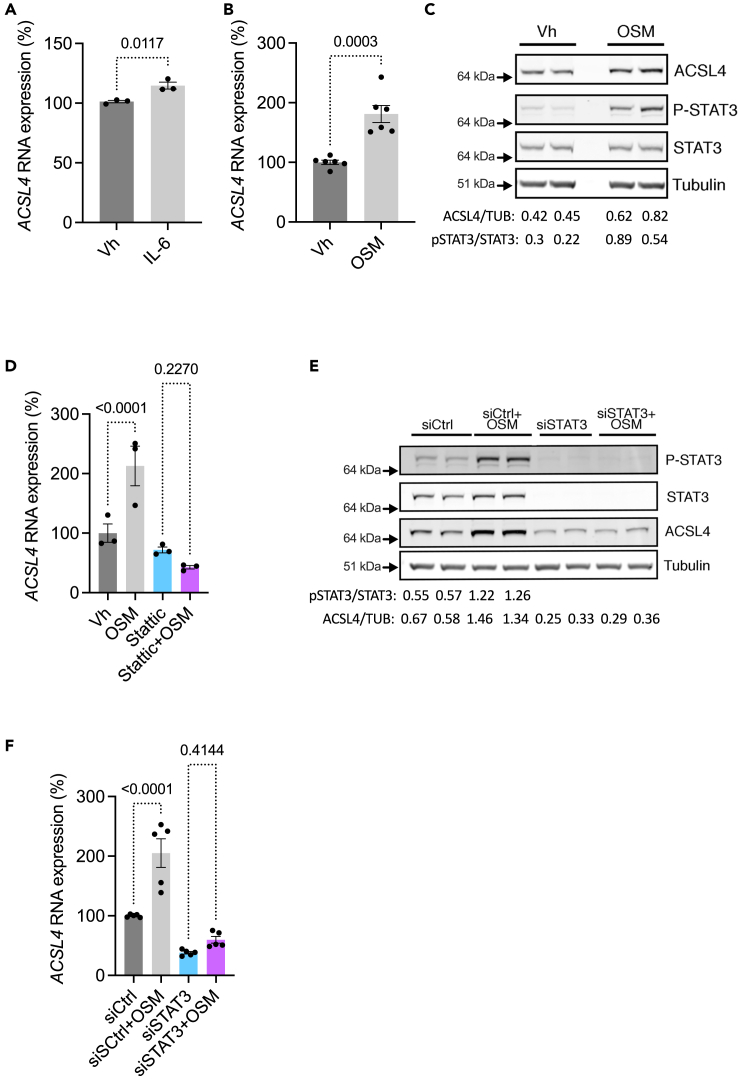
STAT3 signaling promotes the expression of ACSL4 in tubular epithelial cells (A) *ACSL4* transcript levels measured by RT-qPCR in HK-2 cells incubated with 100 ng/mL IL-6 for 24 h (n = 3 replicates per condition). p values were computed with a Student’s t test. Data are represented as mean ± SEM. (B) *ACSL4* transcripts levels measured by RT-qPCR in HK-2 cells incubated with 20 ng/mL OSM for 24 h (n = 6 replicates per condition). p values were computed with a Student’s t test. Data are represented as mean ± SEM. (C) ACSL4, phospho-STAT3 (P-STAT3), STAT3, and tubulin protein levels in HK-2 cells incubated with 20 ng/mL OSM for 24 h. The immunoblot shown is representative of three independent experiments in duplicate. (D) *ACSL4* transcripts levels measured by RT-qPCR in HK-2 cells incubated with 20 ng/mL OSM for 24 h in the presence or absence of 5 μM Stattic (n = 3 replicates per condition). p values were computed with a one-way ANOVA followed by a Šídák multiple comparisons test. Data are represented as mean ± SEM. (E) ACSL4, phospho-STAT3 (P-STAT3), STAT3, and tubulin protein levels in HK-2 cells incubated with 20 ng/mL OSM for 24 h in the presence or absence of siRNA targeting *STAT3* (siSTAT3) or a scrambled siRNA (siCtrl). The immunoblot shown is representative of three independent experiments. (F) *ACSL4* transcripts levels measured in HK-2 cells incubated with 20 ng/mL OSM for 24 h in the presence or absence of siRNA targeting *STAT3* (siSTAT3) or a scrambled siRNA (siCtrl) (n = 5 replicates per condition). p values were computed with a one-way ANOVA followed by a Šídák multiple comparisons test. Data are represented as mean ± SEM.

Having shown that ER stress sets the stage for an autocrine loop of IL-6 secretion and activity in TECs, we hypothesized that STAT3 signaling drives *ACSL4* expression in TECs. To address this, we incubated HK-2 cells with IL-6 and oncostatin M (OSM), another member of the IL-6 family and a well-characterized activator of STAT3, since TECs in culture are poorly responsive to IL-6 (Figure 3H and ref.^30^). In addition, we found that the expression of *OSM* was increased in ER-stressed TECs *in vitro* and *in vivo* (Figure S3). Consistent with its activating effects on STAT3 signaling, IL-6 induced the production of *ACSL4* mRNA in HK-2 cells (Figure 4A). OSM increased STAT3 phosphorylation *in vitro*, which correlated with robust induction of *ACSL4* mRNA and protein expression (Figures 4B–4D).

To determine whether *ACSL4* induction was STAT3 dependent, we inhibited STAT3 signaling by chemical means using Stattic, a non-peptidic selective inhibitor that blocks STAT3 activation, dimerization, and nuclear translocation, or by siRNA-mediated RNA interference to knock down STAT3 expression. Consistent with our hypothesis, chemical inhibition or knockdown of STAT3 completely blunted the upregulation of ACSL4 transcripts in response to OSM (Figures 4E–4G). A recent study showed that ACSL4 is an interferon (IFN)g responsive gene and that STAT1 activation is essential for ACSL4 expression in CD8^+^ T cells.^9^ Although HK-2 cells are highly responsive to IFNg (IFNg induced the target gene *IDO1* thousands of times higher than the control), it failed to induce *ACSL4* expression (Figure S4). Taken together, these results indicate that *ACSL4* is an IL-6 family cytokine responsive gene in TECs under the control of STAT3 signaling.

### ER stress influences the lipidomic composition of the proximal tubule *in vivo*

**Figure 5:**
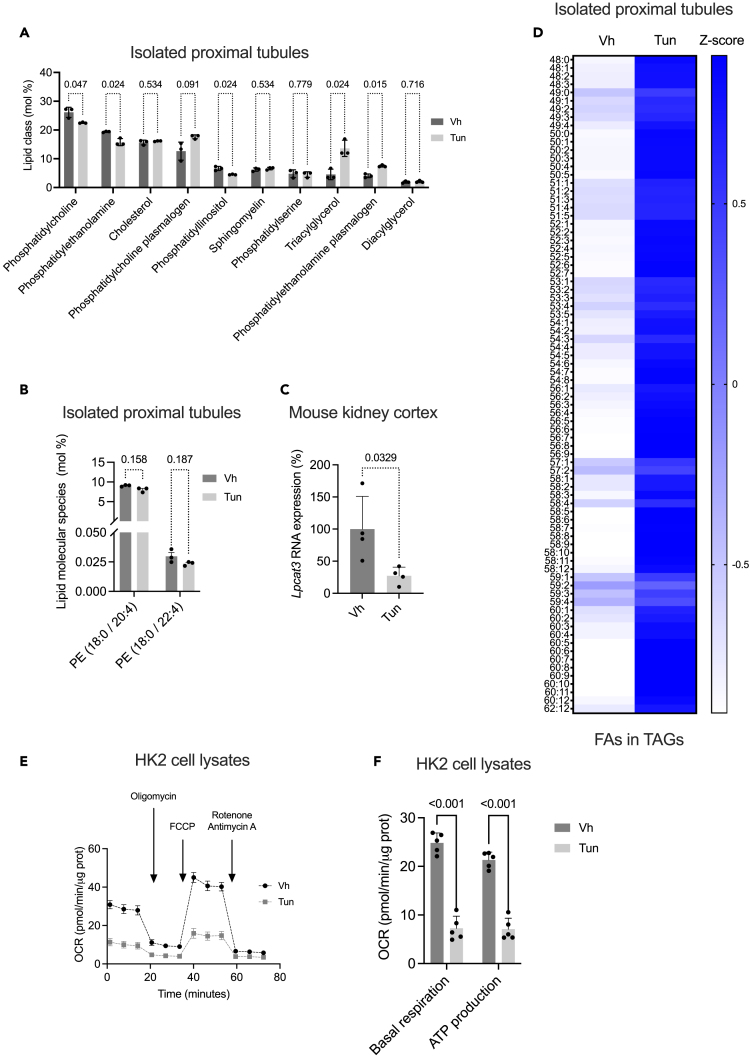
ER stress influences the lipidomic composition of the proximal tubule *in vivo* (A) Absolute quantification of lipid classes in isolated proximal tubules isolate from mouse kidney 48 h after intraperitoneal injection of 1 mg/kg tunicamycin (Tun) or DMSO (Vh) (n = 3 mice per group). *p* values were computed with the FDR approach with the method of Benjamini, Krieger, and Yekiuteli. The y axis indicates the amount of each lipid class normalized to the total membrane lipid amount (excluding CE and TAG) (mol%). Data are represented as mean ± SEM. (B) Contents in PE (18:0/20:4) and PE (18:0/22:4) in isolated proximal tubules from mice 48 h after intraperitoneal injection of 1 mg/kg tunicamycin (Tun) or DMSO (Vh). (n = 3 mice per group). p values were computed with the FDR approach with the method of Benjamini, Krieger, and Yekiuteli, with an FDR<1%. PE, phosphatidylethanolamine. The y axis indicates the relative expression of each lipid species (mol%), which is the percentage of total membrane lipids in the sample. Data are represented as mean ± SEM. (C) *Lpcat3* transcript levels measured by qPCR in kidney cortex of mice 48 h and 96 h after intraperitoneal injection of 1 mg/kg tunicamycin (Tun) or DMSO (Vh) (n = 4 mice per group). p values were computed with a Student t test. Data are represented as mean ± SEM. (D) Heatmap showing the relative composition in fatty acids (FAs) of triacylglycerol (TAG) species in the proximal tubules isolated from mouse kidneys 48 h after intraperitoneal injection of 1 mg/kg tunicamycin (Tun) or DMSO (Vh) (with n = 3 replicates). Each line corresponds to the proportional composition in FAs of TAGs species after autoscaling (i.e., normalizing the data by centering the mean and dividing by the standard deviation of each variable). (E) Oxygen consumption rate (OCR) measured by Seahorse Bioanalyzer in HK-2 cells in response to incubation either with DMSO or with 250 μg/mL tunicamycin (Tun) for 24 h. Arrows indicate the time of the addition of each reagent. Data are represented as mean ± SEM. (F) Quantification of basal respiration and ATP production are shown on the graphs (right panel). p values were computed with the FDR approach with the method of Benjamini, Krieger, and Yekiuteli, with an FDR<1%. Data are represented as mean ± SEM.

We then sought to determine the effect of ER stress on the lipid composition of the proximal tubules, specifically to assess whether increased tubular *ACSL4* expression is associated with enrichment in PE-(18:0/20:4) and PE-(18:0/22:4), the major targets of ferroptosis.^36^ To this end, we manually isolated proximal tubules from mouse kidneys 48 h after injection of 1 mg/kg Tun and subjected the samples to lipidomic profiling by mass spectrometry. This revealed that ER stress altered the lipid composition of proximal tubular cells, with a significant decrease in PEs and PCs compared to vehicle-treated mice (Figures 5A and S5). Furthermore, we did not find a specific enrichment or even a decrease of PE-(C18:0/20:4) and PE-(C18:0/22:4) content in membranes (Figure 5B). Interestingly, lysophosphatidylcholine acyltransferase 3 (*Lpcat3*), which catalyzes the transfer of PUFAs (including AA and AdA) to lysophosphatidylethanolamine (LPE) and lysophosphatidylcholine (LPC) to produce PUFA-PE and PUFA-PC, was profoundly downregulated in the kidneys of Tun-treated mice, which may explain the relative decrease of PEs and PCs in the membrane composition of ER-stressed proximal tubules (Figure 5C). The most pronounced difference was observed for FA storage species such as TAGs, which were significantly enriched in ER-stressed proximal tubules (Figure 5A). Detailed analysis of the degree of saturation of the FAs constituting the TAGs revealed an enrichment of unsaturated FAs in TAGs in proximal tubules from Tun-treated mice compared to vehicle-treated mice, suggesting that in this specific context, PUFAs are preferentially esterified to form TAGs (Figure 5D).

In addition to phospholipid synthesis and storage in TAGs, FAs serve as substrates for mitochondrial beta-oxidation and ATP production. We investigated the metabolic profile of mitochondria from ER-stressed HK-2 cells *in vitro* using the Seahorse Bioanalyzer. In ER-stressed cells, mitochondrial metabolic profiling revealed inhibition of basal and decreased ATP production capacity, indicating a defect in FAO (Figures 5E and 5F).

Taken together, these results indicate that activation of the ER stress response in proximal tubule cells results in a decrease in PE and PC synthesis, an energetic depression consistent with a defect in FAO, and the production of TAGs as a storage species. Our lipidomic analysis of isolated proximal tubules indicates that, despite strong induction of *ACSL4*, there is no enrichment of lipid peroxidation-sensitive PE species, and therefore probably no sensitization to ferroptosis in the context of ER stress. This may be related to the suppression of *LPCAT3* expression.

### ACSL4 does not facilitate tubular epithelial cell death in ferroptotic conditions *in vitro*

Since we have shown that ACSL4 is induced in cultured TECs in response to OSM in a STAT3-dependent manner, we sought to determine whether an increase in ACSL4 activity induced by this inflammatory condition drives epithelial cell ferroptosis *in vitro*. To this end, we tested whether *ACSL4* induced by OSM increases the susceptibility of HK-2 cells to ferroptotic cell death induced by RSL3, a potent and selective GPX4 inhibitor, and whose ferroptotic-promoting activity appears to be particularly efficient in the context of ACSL4-induced membrane remodeling, whereas the activation of ferroptosis by other factors such as erastin, which inhibits the solute carrier family 7 member 11 (SLC7A11), is considered to be independent of ACSL4.^36^^,^^37^^,^^38^^,^^39^

**Figure 6:**
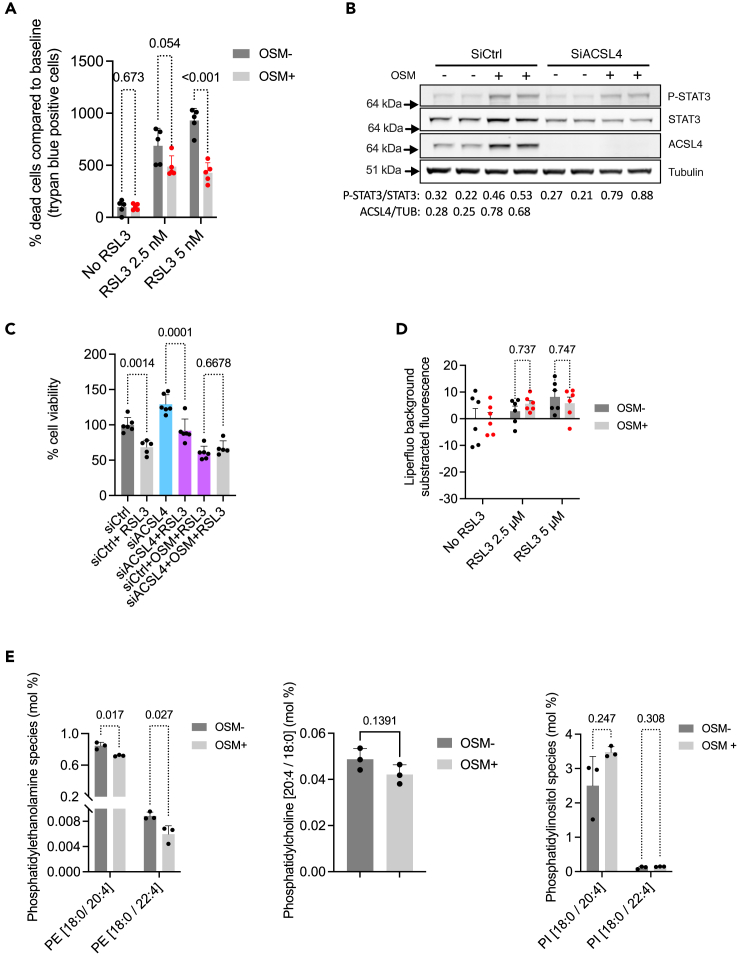
ACSL4 does not facilitate tubular epithelial cell death in ferroptotic conditions *in vitro* (A) Trypan blue staining of HK-2 cells preincubated with 20 ng/mL OSM for 24 h then incubated with or without RSL3 for 24 h (n = 5 replicates per condition). p values were computed with the FDR approach with the method of Benjamini, Krieger, and Yekiuteli, with an FDR<1%. Data are represented as mean ± SEM. (B) Expression of phospho-STAT3 (P-STAT3), STAT3, ACSL4, and tubulin protein levels in HK-2 cells incubated with 20 ng/mL OSM for 24 h in the presence or absence of siRNA targeting *ACSL4* (siACSL4) or a scrambled siRNA (siCtrl). The immunoblot shown is representative of three independent experiments. (C) Viability (MTT assay) of HK-2 cells incubated with 20 ng/mL OSM for 24 h in the presence or absence of siRNA targeting *ACSL4* (siACSL4) or a scrambled siRNA (siCtrl) (n = 6 replicates per condition. p values were computed with a one-way ANOVA followed by a Šídák’s multiple comparisons test. Data are represented as mean ± SEM. (D) Fluorescence intensity of lipid hydroperoxides (Liperfluo staining) in HK-2 cells preincubated with or without 20 ng/mL OSM for 24 h then incubated with RSL3 for 4 h (n = 6 replicates per condition). p values were computed with the FDR approach with the method of Benjamini, Krieger, and Yekiuteli, with an FDR<1%. Data are represented as mean ± SEM. (E) Relative contents in PE (18:0/20:4), PE (18:0/22:4), PC (18:0/20:4), PI (18:0/20:4), and PI (18:0/22:4) in HK-2 cells incubated with 20 ng/mL OSM for 48 h compared to the vehicle condition (n = 3 replicates per condition). p values were computed with the FDR approach with the method of Benjamini, Krieger, and Yekiuteli, with an FDR<1%. PE, phosphatidylethanolamine; PC, phosphatidylcholine; PI, phosphatidylinositol. The y axis indicates the relative expression of each lipid species (mol%), which is the percentage of total membrane lipids in the sample. Data are represented as mean ± SEM.

As expected, RSL3 promoted HK-2 cell death (assessed by trypan blue staining), indicating that these cells are potent targets for ferroptotic cell death (Figure 6A). However, pre-incubation with OSM to induce *ACSL4* expression did not increase RSL3-induced cell death (Figure 6A). Knockdown of *ACSL4* expression by siRNA did not rescue the effect of RSL3 on the viability of OSM-treated cells (Figures 6B and 6C), indicating that OSM-induced ACSL4 activity does not affect the viability of TECs under ferroptotic conditions in our model. We next monitored lipid peroxidation by measuring the fluorescence emitted by LiperFLuo, a fluorescent probe that interacts with lipid hydroperoxides and whose fluorescence reliably reports intracellular sites of lipid hydroperoxide accumulation (Figure S6).^40^ RSL3 caused a dose-dependent increase in the lipid peroxidation signal. However, preincubation with OSM to activate ACSL4 did not affect the signal (Figure 6D).

Since ACSL4 influences the lipid composition required for ferroptosis, we investigated whether elevated ACSL4 alters the membrane lipid composition of TECs. To this end, we performed a global quantitative lipidomics analysis of HK-2 cells incubated 48 h with OSM. OSM significantly reduced the levels of PE, sphingomyelins, and GM3 gangliosides present in the membranes, corresponding to a lipid profile reminiscent of a differentiated tubular epithelium^41^ (Figure S7). More specifically, we found that the levels of ACSL4 substrates such as AA (C20:4) and AdA (22:4) were not enriched in PEs, PCs, and PIs in OSM-treated cells than in vehicle-treated cells (Figure 6E). Importantly, IL-6, which also induces, albeit weakly, ACSL4, did not affect the composition in AA (C20:4) and AdA (22:4) of PEs (Figure S8).

## Discussion

In this study, we show that *ACSL4* is overexpressed by tubular epithelium in a model of ER-stress-induced kidney injury and is upregulated in response to STAT3 signaling in a cell extrinsic manner. Biologically, our findings link IL-6 family cytokines and STAT3 signaling to the regulation of ACSL4 for the first time. In this proinflammatory context, the lipidomic composition of TECs membranes is remodeled, but without enrichment in the species sensitive to lipid peroxidation, which does not render the cell more susceptible to stressors associated with ferroptosis.

The involvement of ACSL4 in the pathophysiology of kidney diseases, particularly AKI, is beginning to be described, and the emerging picture associates it with an injured phenotype and cell death.^17^ For example, it has recently been shown that inhibition of ACSL4 by genetic or chemical means could have a cytoprotective effect in renal I/R injury.^14^^,^^42^ However, ACSL4 is involved not only in the production of PUFA-CoA for incorporation into phospholipids but also in the synthesis of TAGs.^11^ It has recently been shown that TAG synthesis in the hours following I/R injury has cytoprotective properties, since the rapid mobilization of FAs would serve as a substrates for FAO.^18^ Thus, the net consequences of an increase in ACSL4 activity in the context of AKI must be assessed, taking into account both a dynamic aspect of the expression of the enzyme and the presence of partners necessary for the execution of a particular pathway. The same consideration applies to the potential cellular consequences of using inhibitors for kidney protection, since blocking TAG synthesis in the early phase of I/R injury could have deleterious consequences on epithelial regeneration.

In the field of ferroptosis, it has been generally accepted that ACSL4 is universally required for ferroptosis execution, and *ACSL4* knockout cells are widely used to demonstrate that observed cell death phenotypes are due to ferroptosis.^16^ However, recent evidence challenges the view that ACSL4 is a universal ferroptosis regulator and shows that it is only required for ferroptosis under specific circumstances, suggesting that ACSL4 may be dispensable for ferroptosis in some specific contexts.^38^ Our results suggest that it is not sufficient either. Indeed, we show that high expression of ACSL4 in a tissue or cell does not necessarily imply a predisposition to ferroptosis via remodeling of PUFA-containing PEs and should therefore not be considered as a surrogate. It is therefore necessary to understand in a specific context how ACSL4 is induced and how the inducer affects both the enzyme itself for full activity, for example through post-translational modifications.^43^ It is important to consider the activity of other intermediaries on the pathway, involved in the synthesis of PUFA-containing PE, such as LPCAT3, or in the oxidation of C20:4-PEs and C22:4-PEs, such as 15-lipoxygenase (15-LOX). For example, deficiency of *LPCTA3*, which is specific for PE-based substrates, reduces ferroptosis induced by the GPX4 inhibitor RSL3.^44^

The fact that ACSL4 does not predispose to ferroptosis in a specific proinflammatory context, such as ER stress, does not preclude the possibility that it may represent a predisposition to ferroptosis in other situations associated with the production of IL-6 family cytokines and high ACSL4 expression. Under such conditions, ACSL4 activity and ferroptotic cell death may be associated with maladaptive repair after AKI and chronic kidney epithelial injury, which is all the more relevant because IL-6 family cytokines are involved in the pathophysiology of kidney disease and drive tissue remodeling.^30^^,^^31^^,^^32^^,^^33^ Inflammatory cell death such as ferroptosis could be a nidus for subsequent inflammation and production of a variety of cytokines by chronically injured proximal tubular cells, including IL-6 family cytokines that signal via STAT3 activation, likely perpetuating the vicious cycle of maladaptive repair acting in a paracrine manner.^45^ These considerations are consistent with a body of evidence that the most important risk factor for AKI is preexisting CKD, which increases the risk by as much as 10-fold compared with the absence of CKD. The mechanisms by which this occurs are still poorly understood, and from a pathophysiological point of view, it is possible to propose a hypothesis according to which an epithelium engaged in a maladaptive and chronically injured response generates an inflammatory response associated with the activation of STAT3, which induces the expression of ACSL4 in the epithelial microenvironment. Since an increased activity of ACSL4 sensitizes TECs to cell death, it is reasonable to assume that this permanently injured epithelium will offer less resistance when exposed to a ferroptotic stimulus with consequent cell death.

We show that activation of the UPR in cultured TECs represses ACSL4 transcription in a cell-intrinsic manner. On the other hand, these cells secrete IL-6, a potent inducer of ACSL4 expression through Stat3, so there is likely a conflict between an activating and a repressive signal mediated by ER stress. The net result in this particular context is a repression of ACSL4 gene expression, probably associated with an IL-6-Stat3-mediated activating signal that is too weak, in particular due to insufficient concentrations of IL-6 in the culture medium. The situation is probably different *in vivo*, with higher concentrations of Stat3 pathway activators (IL-6 family cytokines) and perhaps a lower intensity of ER stress, which would favor activation of ACSL4 expression. In addition, we demonstrated differential regulation of IL-6 receptors, notably GP130, which mediates IL-6R activity and signal transduction of the IL-6-related cytokines. Indeed, GP130 is highly overexpressed in the renal parenchyma of mice exposed to tunicamycin, which is not the case *in vitro* in HK-2 cells incubated with tunicamycin. The reasons for this difference are not known, but it is likely that the renal microenvironment plays a critical role in regulating GP130 expression in the context of ER stress.

Many paracrine signals are generated during ER stress by affected cells, including those of the IL-6 family (e.g., IL-11, IL-27, CTF-1, CLCF1), which activate Stat3 signaling, among others. Our *in vitro* results clearly show that Stat3 is required for ACSL4 expression by IL-6 family cytokines.

In conclusion, we identified ACSL4 as a cardinal feature of ER-stress-associated AKI that is regulated in a non-cell autonomous manner by IL-6 family cytokines. A proinflammatory microenvironment associated with STAT3 activation leads to ACSL4 expression in TECs and membrane lipidome remodeling without sensitization to ferroptosis. Thus, ACSL4 is not sufficient for ferroptosis sensitization, underscoring the importance of the biological context associated with the study model.

### Limitations of the study

Our *in vivo* results suggest that the STAT3 pathway is active, but we have not formally demonstrated the role of Stat3 *in vivo* in regulating ACSL4 expression the tubular epithelium. This would require the development of a Stat3-dependent tissue deletion model in the tubule. *In vivo* validation by generating tubule-specific Stat3-knockout mice is lacking in our study, and to our knowledge the available model is a partial deletion.^30^

## STAR★Methods

### Key resources table

**Table:** 

REAGENT or RESOURCE	SOURCE	IDENTIFIER
**Antibodies**		

Goat monoclonal Anti-HSPA5 (BIP, GRP78)	Santa Cruz Biotechnology	Cat # sc1050; RRID: AB_631616
Rabbit monoclonal Anti-ACSL4	Sigma-Aldrich	Cat # HPA005552; RRID: AB_1078774
Mouse STAT3	Cell Signaling	CS9139; RRID: AB_331757
Rabbit p-STAT3 (Tyr705)	Cell Signaling Technology	Cat # CS9145; RRID: AB_2491009
Mouse Tubulin	Millipore Sigma	Cat# T9026; RRID: AB_477593

**Chemicals, peptides, and recombinant proteins**		

Tunicamycin from Streptomyces Sp	Sigma-Aldrich	Cat# T7765, Cas 11089-65-9
Thapsigargin	Sigma-Aldrich	Cat# T9033, Cas 67526-95-8
DL-Dithiothréitol	Sigma-Aldrich	Cat# 646563, Cas 3483-12-3
Brefeldin A	Sigma-Aldrich	Cat# B5938, Cas 2035-15-6
RSL3	MedChemExpress	Cat# HY-100218A, Cas 1219810-16-8
Stattic	MedChemExpress	Cat# HY-13818, Cas 19983-44-9
Actinomycin D	Sigma-Aldrich	Cat# A9415, Cas 50-76-0
Cyclosporine	Sigma-Aldrich	Cat# 30024, Cas 59865-13-3
IRE1 Inhibitor IV, KIRA6	Millipore	Cat# 532281, Cas 53-2281
Human OSM (rhOncostatin M)	R&D System	Cat# 295-OM/CF
Human recombinant IL-6	Sigma-Aldrich	Cat# GM338
Liperfluo	Doninjo Laboratories	Cat# L248-10 Cas 1448846-35-2
MTT (3-(4,5-dimethylthiazol-2-yl)-2,5-diphenyltetrazolium bromide)	Sigma-Aldrich	Cat# M2128, Cas 298-93-1

**Critical commercial assays**		

TUNEL Assay Kit – BrdU-Red	Abcam	Cat# Ab66110
Thermo Scientific Pierce BCA Protein Assay Kits	Thermo Fisher Scientific	Cat# 10741395
RNeasy Mini Kit	QIAGEN	Cat# 74106
Hight Capacity cDNA Reverse Transcription Kit	Applied Biosytem	Cat# 4368814
AB Solute Blue qPCR SYBR Green ROX Mix	ThermoScientific	Cat# AB-4162/B
Dako EnVision r+ Dual Link System-HRP	Agilent	Cat# K4065
Mycoalert Mycoplasma Detection Kit	Lonza	Cat# LT07-318

**Deposited data**		

Lipidomic standard reporting checklist	This paper	https://doi.org/10.5281/zenodo.10908592
Lipidomic raw data on Mendeley	This paper	Mendeley Data, V1, https://doi.org/10.17632/w8kst7mpr8.1

**Experimental models: Cell lines**		

HK-2 Cell line	ATCC/LGS	ATCC-CRL-2190

**Experimental models: Organisms/strains**		

Mouse: C57BL/6	Charles River Laboratories	N/A

**Oligonucleotides**		

AllStars Negative Control siRNA	QIAGEN	SI03650318
Hs_ACSL4_1	QIAGEN	SI00063980
Hs_ACSL4_3	QIAGEN	SI00063994
Hs_STAT3_3	QIAGEN	SI00048377
Hs_STAT3_4	QIAGEN	SI00048384
Hs_STAT3_7	QIAGEN	SI02662338
Hs_STAT3_8	QIAGEN	SI02662898
***Mouse Acsl1*** F 5’ ATC TGG TGG AAC GAG GCA AG 3’ R 5’ TCC TTT GGG GTT GCC TGT AG 3’	This paper	N/A
***Mouse Acsl3*** F 5’ AAC TTT GGG TTG CTG AGT GGA 3’ R 5’ CAA GTG TAT CGC AGC CAG GA 3’	This paper	N/A
***Mouse Acsl4*** F 5’ CTT CCT CTT AAG GCC GGG AC 3’ R 5’ TCT CTT TGC CAT AGC GTT TTT AGA 3’	This paper	N/A
***Mouse Acsl5*** F 5’ AAT GTG TTC AAA GGC TAC CTA AAG GAC CC 3’ R 5’ GCG ACC AAT GTC CCC AGT GTG A 3’	This paper	N/A
***Mouse Ddit3*** F 5’ CAA CAG AGG TCA CAC GCA CA 3’ R 5’ GGC ACT GAC CAC TCT GTT TC 3’	This paper	N/A
***Mouse HspA5*** F 5’ CAC CAG GAT GCG GAC ATT GA 3’ R 5’ AGG GCC TCC ACT TCC ATA GA 3’	This paper	N/A
***Mouse Il-6*** F 5’ TAC CCC AAC TTC CAA TGC TC 3’ R 5’ GGT TTG CCG AGT AGA CCT CA 3’	This paper	N/A
***Mouse Rpl13a*** F 5’ CCC TAT GAC AAG AAA AAG CGG A 3’ R 5’ TTT CCT TCC GTT TCT CCT CCA G 3’	This paper	N/A
***Mouse Slc7A11*** *F 5’* ACT GTT CGG TCG TGA CTT CC 3’ *R 5’* AAT ACG GAG CCT TCC ACG AG 3’	This paper	N/A
***Mouse Gpx4*** F 5’ GCA ACC AGT TTG GGA GGC AGG AG 3’ R 5’ CCT CCA TGG GAC CAT AGC GCT TC 3’	This paper	N/A
***Mouse Fsp1*** F 5’ GGC CTC TGC TTC ATG GAG TT 3’ R 5’ CTG GCT AGC AAT GGT GCT CT 3’	This paper	N/A
**Mouse Osm** F 5’ AAG GAA CAC TGA TCT GGG CG R 5’ TTG CAC CAC AGG TTC CCA TT	This paper	N/A
***Mouse IL6ST*** GCCAGAGTCCTTCAGAGAG TGGTCCTTAGCCACTCCTTC	This paper	N/A
***Mouse IL6R*** AGGGAATGAGCCTGTGAACA AGGCCACTCAGTCAAACGTA	This paper	N/A
**Human *Acsl4*** F 5’ TTC TCA TTC TTC CCA ACT TGC CT 3’ R 5’ GGC AGA TTG GGG GTG CAA AT 3’	This paper	N/A
***Human Hspa5*** F 5’ GGT GAA AGA CCC CTG ACA AA 3’ R 5’ GTC AGG CGA TTC TGG TCA TT 3’	This paper	N/A
***Human IL-6*** F 5’ CCA GGA GAA GAT TCC AAA GAT GTA 3’ R 5’ CGT CGA GGA TGT ACC GAA TTT 3’	This paper	N/A
***Human Ido1*** F 5’ GTG AAA GCT CTG GTC TCC CT 3’ R 5’ TCA GTG CCT CCA GTT CCT TT 3’	This paper	N/A
***Human spliced Xbp1*** F 5’ TGC TGA GTC CGC AGC AGG TG 3’ R 5’ GCT GGC AGG CTC TGG GGA AG 3’	This paper	N/A
***Human Slc7a11*** F 5’ GAG CAC GAT GCA TAC ACA GG 3’ R 5’ TCC CTG CAG GTA ACC TCC TT 3’	This paper	N/A
***Human OSM*** F 5’ CAT GGG GGT ACT GCT CAC AC 3’ R 5’ ATT GAG GGT CTG CAG GAA GC 3’	This paper	N/A
***Human IL6ST*** GCAAAGCAAAACGTGACACC AATTATGTGGCGGATTGGGC	This paper	N/A
qPCR Primers and antibodies Mouse Acsl1 F 5’ ATC TGG TGG AAC GAG GCA AG 3’ R 5’ TCC TTT GGG GTT GCC TGT AG 3’ Acsl3 F 5’ AAC TTT GGG TTG CTG AGT GGA 3’ R 5’ CAA GTG TAT CGC AGC CAG GA 3’ Acsl4 F 5’ CTT CCT CTT AAG GCC GGG AC 3’ R 5’ TCT CTT TGC CAT AGC GTT TTT AGA 3’ Acsl5 F 5’ AAT GTG TTC AAA GGC TAC CTA AAG GAC CC 3’ R 5’ GCG ACC AAT GTC CCC AGT GTG A 3’ Ddit3 F 5’ CAA CAG AGG TCA CAC GCA CA 3’ R 5’ GGC ACT GAC CAC TCT GTT TC 3’ HspA5 F 5’ CAC CAG GAT GCG GAC ATT GA 3’ R 5’ AGG GCC TCC ACT TCC ATA GA 3’ Il-6 F 5’ TAC CCC AAC TTC CAA TGC TC 3’ R 5’ GGT TTG CCG AGT AGA CCT CA 3’ Rpl13a F 5’ CCC TAT GAC AAG AAA AAG CGG A 3’ R 5’ TTT CCT TCC GTT TCT CCT CCA G 3’ Slc7A11 F 5’ ACT GTT CGG TCG TGA CTT CC 3’ R 5’ AAT ACG GAG CCT TCC ACG AG 3’ Gpx4 F 5’ GCA ACC AGT TTG GGA GGC AGG AG 3’ R 5’ CCT CCA TGG GAC CAT AGC GCT TC 3’ Fsp1 F 5’ GGC CTC TGC TTC ATG GAG TT 3’ R 5’ CTG GCT AGC AAT GGT GCT CT 3’ Osm F 5’ AAG GAA CAC TGA TCT GGG CG R 5’ TTG CAC CAC AGG TTC CCA TT IL6ST GCCAGAGTCCTTCAGAGAGTGGTCCTT AGCCACTCCTTC IL6R AGGGAATGAGCCTGTGAACAAGGCCA CTCAGTCAAACGTA qPCR Primers and antibodies Mouse Acsl1 F 5’ ATC TGG TGG AAC GAG GCA AG 3’ R 5’ TCC TTT GGG GTT GCC TGT AG 3’ Acsl3 F 5’ AAC TTT GGG TTG CTG AGT GGA 3’ R 5’ CAA GTG TAT CGC AGC CAG GA 3’ Acsl4 F 5’ CTT CCT CTT AAG GCC GGG AC 3’ R 5’ TCT CTT TGC CAT AGC GTT TTT AGA 3’ Acsl5 F 5’ AAT GTG TTC AAA GGC TAC CTA AAG GAC CC 3’ R 5’ GCG ACC AAT GTC CCC AGT GTG A 3’ Ddit3 F 5’ CAA CAG AGG TCA CAC GCA CA 3’ R 5’ GGC ACT GAC CAC TCT GTT TC 3’ HspA5 F 5’ CAC CAG GAT GCG GAC ATT GA 3’ R 5’ AGG GCC TCC ACT TCC ATA GA 3’ Il-6 F 5’ TAC CCC AAC TTC CAA TGC TC 3’ R 5’ GGT TTG CCG AGT AGA CCT CA 3’ Rpl13a F 5’ CCC TAT GAC AAG AAA AAG CGG A 3’ R 5’ TTT CCT TCC GTT TCT CCT CCA G 3’ Slc7A11 F 5’ ACT GTT CGG TCG TGA CTT CC 3’ R 5’ AAT ACG GAG CCT TCC ACG AG 3’ Gpx4 F 5’ GCA ACC AGT TTG GGA GGC AGG AG 3’ R 5’ CCT CCA TGG GAC CAT AGC GCT TC 3’ Fsp1 F 5’ GGC CTC TGC TTC ATG GAG TT 3’ R 5’ CTG GCT AGC AAT GGT GCT CT 3’ Osm F 5’ AAG GAA CAC TGA TCT GGG CG R 5’ TTG CAC CAC AGG TTC CCA TT IL6ST GCCAGAGTCCTTCAGAGAG TGGTCCTTAGCCACTCCTTC IL6R AGGGAATGAGCCTGTGAACA AGGCCACTCAGTCAAACGTA qPCR Primers and antibodies Mouse Acsl1 F 5’ ATC TGG TGG AAC GAG GCA AG 3’ R 5’ TCC TTT GGG GTT GCC TGT AG 3’ Acsl3 F 5’ AAC TTT GGG TTG CTG AGT GGA 3’ R 5’ CAA GTG TAT CGC AGC CAG GA 3’ Acsl4 F 5’ CTT CCT CTT AAG GCC GGG AC 3’ R 5’ TCT CTT TGC CAT AGC GTT TTT AGA 3’ Acsl5 F 5’ AAT GTG TTC AAA GGC TAC CTA AAG GAC CC 3’ R 5’ GCG ACC AAT GTC CCC AGT GTG A 3’ Ddit3 F 5’ CAA CAG AGG TCA CAC GCA CA 3’ R 5’ GGC ACT GAC CAC TCT GTT TC 3’ HspA5 F 5’ CAC CAG GAT GCG GAC ATT GA 3’ R 5’ AGG GCC TCC ACT TCC ATA GA 3’ Il-6 F 5’ TAC CCC AAC TTC CAA TGC TC 3’ R 5’ GGT TTG CCG AGT AGA CCT CA 3’ Rpl13a F 5’ CCC TAT GAC AAG AAA AAG CGG A 3’ R 5’ TTT CCT TCC GTT TCT CCT CCA G 3’ Slc7A11 F 5’ ACT GTT CGG TCG TGA CTT CC 3’ R 5’ AAT ACG GAG CCT TCC ACG AG 3’ Gpx4 F 5’ GCA ACC AGT TTG GGA GGC AGG AG 3’ R 5’ CCT CCA TGG GAC CAT AGC GCT TC 3’ Fsp1 F 5’ GGC CTC TGC TTC ATG GAG TT 3’ R 5’ CTG GCT AGC AAT GGT GCT CT 3’ Osm F 5’ AAG GAA CAC TGA TCT GGG CG R 5’ TTG CAC CAC AGG TTC CCA TT IL6ST GCCAGAGTCCTTCAGAGAG TGGTCCTTAGCCACTCCTTC IL6R AGGGAATGAGCCTGTGAACA AGGCCACTCAGTCAAACGTA ***Human IL6R*** AGCAGAGGAAGGAGAGGAGA TGGGAGGTGGAGAAGAGAGA	This paper	N/A
***Human Rpl13a*** F 5’ CCT GGA GGA GAA GAG GAA AGA GA 3’ R 5’ GAG GAC CTC TGT GTA TTT GTC AA 3’	This paper	N/A

**Software and algorithms**		

GraphPad Prism 10 for Mac	GraphPad Software	Version 10.0.2
ImageJ	https://imagej.nih.gov/ij/	Version 1.53.t

**Other**		

QuantStudio 7 Flex Real-Time PCR System	Applied Biosytems	N/A
Tecan Safire r plate reader	Tecan	

### Resource availability

#### Lead contact

Further information and requests for resources and reagents should be directed to and will be fulfilled by the lead contact, Nicolas Pallet: Nicolas.pallet@aphp.fr.

#### Materials availability

This study did not generate new unique reagents.

#### Data and code availability

• The lipidomic standard reporting checklist (https://lipidomicstandards.org/) is available at the https://doi.org/10.5281/zenodo.10908592, and Lipidomic raw data are available as Mendeley Data, V1, https://doi.org/10.17632/w8kst7mpr8.1. All are publicly available as of the date of publication.
• This paper does not report original code.
• Any additional information required to reanalyze the data reported in this paper is available from the lead contact upon request.

### Experimental model and study participant details

#### *In vivo* animal studies

12 weeks-old C57BL/6J background male mice (Charles River laboratories). Males are more susceptible to toxic injuries. The animals were kept at CEF (Centre d’Explorations Fonctionnelles) of the Cordeliers Research Center, Agreement no. A75-06-12). Animals were fed *ad libitum* and housed at 25°C in a 12-hour light cycle. All animal experimentations were conducted in accordance with the institutional guidelines and the recommendations for the care and use of laboratory animals put forward by the Directive 2010/63/EU revising Directive 86/609/EEC on the protection of animals used for scientific purposes (project has been approved by a user establishment's ethics committee and the Project Authorization: number 21927).

#### Cell lines

HK2 cells (ATCC/LGC Standards (lot number 60352186) were established by transduction with human papilloma virus (HPV 16) E6/E7 genes from a primary proximal tubule cells culture from normal male adult human kidney cortex. HK-2 cells are cultured at 37°C in Dulbecco's Modified Eagle Medium (DMEM) containing 5 μg/mL insulin, 10 μg/mL human apotransferrin, 500 ng/mL hydrocortisone, 10 ng/mL Epithelial growth factor, 6.5 ng/mL triiodothyronin, 5 ng/mL sodium selenite, 1% fetal calf serum, 25 IU/mL penicillin, 25 μg/mL streptomycin and 10 mM HEPES buffer. These cells lines are Mycoplasm free.

### Method details

#### Animal experiments

12 weeks-old C57BL/6 background male mice (Charles River laboratories) were intraperitoneally injected with tunicamycin (Sigma-Aldrich, T7765) (1 mg/kg) or vehicle (DMSO) at day 0 and were euthanized 2- and 4-days post-injection (n=6 mice per condition).

#### Proximal tubule isolation

Isolation of proximal convoluted tubules was performed according to localization in the kidney (cortex vs. medulla) and well-defined morphologic characteristics under binocular loupes after kidney treatment with Liberase (Sigma-Aldrich).^46^ Tubule length was measured using Visilog software (Noesis), and pools of 150 mm (approximately 150 segments) were transferred to 500 μl of PBS, rinsed 3 times, centrifuged (600g, 5 minutes), and resuspended in 150 mM ammonium bicarbonate. Samples were frozen in liquid nitrogen and stored at -80°C until lipid extraction (n=3 mice per group).

#### Cells

Normal human kidney epithelial cells of proximal origin (HK-2) were purchased from ATCC/LGC Standards (lot number 60352186), and cultured according to previously published method.^47^ HK-2 is a cell line derived from primary proximal tubule cells. HK-2 cells are cultured in Dulbecco's Modified Eagle Medium (DMEM) containing 5 μg/mL insulin, 10 μg/mL human apotransferrin, 500 ng/mL hydrocortisone, 10 ng/mL epithelial growth factor, 6.5 ng/mL triiodothyronin, 5 ng/mL sodium selenite, 1% fetal calf serum, 25 IU/mL penicillin, 25 μg/mL streptomycin and 10 mM HEPES buffer. These cells lines are Mycoplasm free (Mycoalert Mycoplasma Detection Kit, Lonza).

Tunicamycin, thapsigargin, dithiotreitol, brefeldin A, actinomycin D, cyclosporine, DMSO and IL-6 were purchased from Sigma Aldrich. OSM was purchased from R&D systems. KIRA6 was purchased from Millipore. RSL3 and Stattic were purchased from MedChem Express.

#### RNA extraction and real-time quantitative polymerase chain reaction (RT-qPCR)

Total RNA was extracted using the RNeasy Mini Kit® (Qiagen) according to the manufacturer's protocol. Transcript expression levels were quantified by SYBR green RT-qPCR using the QuantStudio™ 7 Flex Real-Time PCR System (Applied Biosystems). Vehicle-treated samples were used as controls, and fold changes for each gene tested were normalized to the ribosomal protein L13A (RPL13A) housekeeping gene. Relative expression levels were calculated using the 2-ΔΔCT method.^48^

#### Protein extraction and immunoblotting

Cells were washed in PBS and incubated for 15 minutes at 4°C and in NADOC (0.5% Tritonx-100, 0.5% sodium deoxycholate, 50 mM Tris, and 150 mM NaCl) lysis buffer (Thermo Fisher Scientific) with protease (HaltTM Protease Inhibitor Cocktail 100X, Thermo Fisher Scientific) and phosphatase inhibitors (HaltTM Phosphatase Inhibitor Cocktail 100X, Thermo Fisher Scientific). The extracts were centrifuged at 14,000g for 15 minutes. Protein concentrations in the supernatant were measured using a Pierce BCA protein assay kit (Thermo Fisher Scientific) and a Tecan Safire® plate reader. Protein extracts (25 μg) were resolved by 4-12% SDS-PAGE (Invitrogen) and transferred to nitrocellulose membranes (iBlot, Invitrogen). Membranes were blocked with SEABLOCK blocking buffer (Thermo-Scientific) for 1 hour at room temperature and then incubated overnight at 4°C with primary antibodies diluted in blocking buffer. After washing in PBS-Tween buffer, the membranes were incubated with secondary antibodies conjugated to IRDye fluorophores. The infrared signal of the membranes was detected using an Odyssey detection system (Li-Cor biosciences).

#### Immunohistochemistry

Kidney slices were fixed in alcohol-formalin-acetic acid, dehydrated in ethanol and xylene, embedded in paraffin, and cut into 3 mm sections. Samples were then deparaffinized, rehydrated, and heated at 97°C for 20 minutes in citrate buffer. Endogenous peroxidase was inactivated by incubation in 0.3% H2O2 for 10 minutes at room temperature. Sections were incubated with PBS containing anti-HSPA5 (BiP, GRP78, sc-1050, Santa Cruz Biotechnology), anti-ACSL4 (Sigma HPA005552). This antibody has been validated in the Human Protein Atlas project (https://www.proteinatlas.org/ENSG00000068366-ACSL4/summary/antibody). pSTAT3 immunohistochemistry was performed using TBS instead of PBS and p-STAT3 Tyr705 antibody (Cell Signaling CS9145). Subsequently, the sections were incubated with anti-rabbit or anti-goat antibodies conjugated to peroxydase-labeled polymer and visualized with a peroxydase kit (Dako EnVision®+ Dual Link System-HRP (DAB+), Agilent). Finally, tissue sections were counterstained with hematoxylin. The samples were visualized using a Nikon Eclipse Ti microscope.

#### siRNA transfections

Transient inactivation of *ACSL4* and *STAT3* was achieved using synthetic small interfering RNAs (siRNAs) designed and purchased from Qiagen and transfected using Lipofectamine 3000 (Invitrogen) according to the manufacturer's protocol. Two different siRNA against the same target were transfected for *ACSL4* mRNA: Hs_ACSL4_1 SI00063980, Hs_ACSL4_3, SI00063994, and 4 were transfected for *STAT3:* Hs_STAT3_3 SI00048377, Hs_STAT3_4 SI00048384, Hs_STAT3_7 SI02662338, and Hs_STAT3_8 SI02662898. AllStars Negative Control siRNA (5′-AACGAUGACACGAACACACTT-3′) has no homology to any known mammalian gene and was validated using Affymetrix GeneChip arrays and a variety of cell-based assays to ensure minimal non-specific effects on gene expression and phenotype. Cells were incubated with siRNA for at least 48 hours prior to experiments.

#### Cell death assays

##### MTT (3-(4,5-dimethylthiazol-2-yl)-2,5-diphenyltetrazolium bromide) assay

Cytotoxicity was determined using the CellTiter 96® Non-Radioactive Cell Proliferation Assay (MTT, Promega). Briefly, cells were seeded in 24-well tissue culture plates at 10,000 to 15,000 cells/well and incubated overnight. The exponentially growing cells were then exposed to different drug concentrations (or different compounds) for 24 hours. Cell viability was determined by exposing the cells to MTT tetrazolium salt for 3 hours at 37°C, and formazan formation was measured at 560 nm using a microplate reader after resuspension in DMSO. The percentage of cell viability was determined by dividing the signal in treated cells to the basal conditions. All values are the average of at least three independent experiments, each performed in triplicate or more.

##### Trypan blue exclusion

Cells were seeded in 6-well plates and treated with appropriate stimuli, and dead cells were counted at various times using the trypan blue dye exclusion assay. Medium containing floating cells was collected and centrifuged. Adherent cells were trypsinized and centrifuged. Pellets of floating and detached cells were resuspended in PBS, spread on Mallassez cells, and counted with a phase contrast microscope. The percentage of cell death was determined by dividing the number of dead cells (stained blue) by the total number of cells and expressed as the percentage of dead cells under basal conditions. All values are the average of at least three independent experiments, each performed in triplicate or more.

#### ELISA

Subconfluent cells were grown in 12-wells plates for the indicated times under the indicated conditions. Secretion of IL-6 was quantified in the cell culture supernatant using the Quantikine® human IL6 immunoassay (RD Systems), respectively, according to the manufacturer’s protocol.

#### Liperfluo staining

Cells were seeded in a 96-well plate at a concentration of 15000/well. The next day, cells were prestained with 10 μM LiperFluo (Doninjo) for 30 minutes at 37°C and then treated with 2.5 or 5 nM RSL3. After incubation, fluorescence was measured in a Tekan fluorometer with excitation and emission wavelengths of 488 nm and 535 nm, respectively. All values are the average of at least three independent experiments, each performed in triplicate or more.

#### Mitochondrial activity measurements

For oxygen consumption rate measurements using the Seahorse bioanalyzer, HK-2 cells were seeded at a density of 6 × 10^4^ cells per well in a collagen-coated XFe96 cell culture microplate. Twenty hours after plating, cells were incubated with Tunicamycin for 24 hours, and mitochondrial activity was assessed. Prior to measurement, cells were balanced for 1 h in unbuffered XF assay media (Agilent Technologies) supplemented for OCR analysis with 2 mM glutamine, 10 mM glucose, and 1 mM sodium pyruvate. For OCR measurements, compounds were injected during the assay at the following final concentrations Oligomycin (ATP synthase inhibitor to measure respiration associated with cellular ATP production; 1 μM), FCCP (uncoupling agent to measure maximal respiratory capacity; 1 μM), Rotenone and Antimycin A (ETC inhibitors to measure non-mitochondrial respiration; 1 μM).

#### Electron microscopy

For electron microscopy of mouse kidneys, the samples were fixed in a solution of 2% glutaraldehyde 0.1 M sodium cacodylate, post-fixed in 1% OsO4, and dehydrated in alcohol. They were then processed for flat embedding in Epon 812 and observed in a Zeiss CEM 902 electron microscope.

#### TUNEL staining

DNA fragmentation of nuclei in mouse kidneys were detected using the Terminal deoxynucleotidyl Transferase Biotin-dUTP Nick End Labeling (TUNEL) method (TUNEL Assay Kit - BrdU-Red (Abcam, ab66110) using manufacturer recommendation. The samples were visualized using a Nikon Eclipse Ti microscope.

#### Lipidomic profiling

For Lipidomics analysis, 150 mm of isolated tubules or 100,000 HK-2 cells were spiked with 4.56 μL of internal standard lipid mixture containing 500 pmol of Chol-d6, 100 pmol of Chol-16:0-d7, 100 pmol of DAG 17:0-17:0, 50 pmol of TAG 17:0-17:0-17:0, 100 pmol of SM 18:1;2-12:0, 30 pmol of Cer 18:1;2-12:0, 30 pmol of GalCer 18:1;2-12:0, 50 pmol of LacCer 18:1;2-12:0, 300 pmol of PC 17:0-17:0, 50 pmol of PE 17:0-17:0, 50 pmol of PI 16:0-16:0, 50 pmol of PS 17:0-17:0, 30 pmol of PG 17:0-17:0, 30 pmol of PA 17:0-17:0, 40 pmol of Gb3 18:1;2-17:0, 25 pmol of GM3 18:1;2-18:0-d5, 25 pmol of GM2 18:1;2-18:0-d9, 25 pmol of GM1 18:1;2-18:0-d5, 30 pmol of LPA 17:0, 30 pmol of LPC 12:0, 30 pmol of LPE 17:1 and 30 pmol of LPS 17:1 and subjected to lipid extraction at 4°C, as described elsewhere.^41^ Briefly, the sample was dissolved in 200 μL of 155 mM ammonium bicarbonate and then extracted with 1 mL of chloroform-methanol (10:1) for 2 hours. The lower organic phase was collected, and the aqueous phase was re-extracted with 1 mL of chloroform-methanol (2:1) for 1 hour. The lower organic phase was collected and evaporated in a SpeedVac vacuum concentrator. Lipid extracts were dissolved in 100 μL of infusion mixture consisting of 7.5 mM ammonium acetate dissolved in propanol:chloroform:methanol [4:1:2 (vol/vol)].

Samples were analyzed by direct infusion in a QExactive mass spectrometer (Thermo Fisher Scientific) equipped witha TriVersa NanoMate ion source (Advion Biosciences). 5 μL of sample were infused with gas pressure and voltage set to 1.25 psi and 0.95 kV, respectively. DAG, TAG and CE species were detected in the 10:1 extract, by positive ion mode FTMS as ammonium aducts by scanning m/z= 580–1000 Da, at R_m/z=200_=280 000 with lock mass activated at a common background (m/z=680.48022) for 30 seconds. Every scan is the average of 2 micro-scans, automatic gain control (AGC) was set to 1E6 and maximum ion injection time (IT) was set to 50ms. For FA profiling of DAG and TAG, a parallel reaction monitoring was performed with an inclusion list of m/z=580-1000 Da, at NCE of 20 and R_m/z=200_=17500 for 90 seconds. Every scan is the average of 2 micro-scans, automatic gain control (AGC) was set to 1E5 and maximum ion injection time (IT) was set to 64 ms.

PC, PCO, Cer, GlcCer, LPC and LPCO were detected as acetate adducts while PG, PE, PEO, LPE and LPEO were detected as deprotonated adducts in the 10:1 extract, by negative ion mode FTMS, after polarity switch by scanning m/z= 420–1050 Da, at R_m/z=200_=280 000 with lock mass activated at a common background (m/z=529.46262) for 30 seconds. Every scan is the average of 2 micro-scans, automatic gain control (AGC) was set to 1E6 and maximum ion injection time (IT) was set to 50ms. For FA profiling of PC, PCO, PE, PEO and PG, a parallel reaction monitoring was performed with an inclusion list of m/z=590-940 Da, at NCE of 35 and R_m/z=200_=17500 for 72 seconds. Every scan is the average of 2 micro-scans, automatic gain control (AGC) was set to 1E5 and maximum ion injection time (IT) was set to 64 ms.

LacCer and Gb3 were detected as protonated ions and Gb4 was detected as ammoniated adduct in the 2:1 extract in positive ion mode FTMS by scanning m/z = 800–1,600 Da, at R_m/z=200_ = 280,000 with lock mass activated at a common background (m/z = 1,194.8179) for 30 s. GM1, GM2, andGM3 were detected as deprotonated ions in the 2:1 extract in negative ion mode after polarity switch in FTMS by scanning m/z = 1,100–1,650 Da, at R_m/z=200_= 280,000 with lock mass activated at a common background (m/z = 1,175.7768) for 30 s. Every scan is the average of two micro-scans, AGC was set to 1E6 and IT was set to 50 ms in both polarities.

PA, PI, PS, LPA, and LPS were detected as deprotonated ions in the 2:1 extract in negative ion mode in FTMS by scanning m/z = 400–1,100 Da, at R_m/z=200_ = 280,000 with lock mass activated at a common background (m/z = 529.4626) for 30 s. Every scan is the average of two micro-scans, AGC was set to 1E6 and IT was set to 50 ms. For FA profiling of PA, PI and PS, a parallel reaction monitoring was performed with an inclusion list of m/z=590-940 Da, at NCE of 35 and R_m/z=200_=17500 for 84 seconds. Every scan is the average of 2 micro-scans, automatic gain control (AGC) was set to 1E5 and maximum ion injection time (IT) was set to 64 ms.

All data was acquired in centroid mode. All lipidomics data were analyzed with the lipid identification software, LipidXplorer.^49^ Tolerance for MS and identification was set to 2 ppm. Data post-processing and normalization to internal standards were done manually in Excel. Data analysis was performed in the MetaboAnalyst 5.0 software.^50^ Samples were normalized by sum and each lipid amount was adjusted by autoscaling.

### Quantification and statistical analysis

Graphs and statistical analyses were generated using GraphPad Prism 9 software (GraphPad Software, Inc.). Data are presented as mean ± SEM. Unpaired 2-sample t-tests were used to determine a significant difference between two groups and were 2-tailed. Multiple comparisons were performed with one-way ANOVA followed by a Dunnett’s or a Šídák multiple comparison test. For multiple comparisons between groups, the False Discovery (Rate FDR) approach was used (method of Benjamini Krieger and Yekiuteli), with a FDR (Q)<1%. A p-value < 0.05 was considered a statistically significant difference.
