# Supplementary material for: STAT3 drives the expression of ACSL4 in acute kidney injury
Source: iScience. 2024 Apr 16;27(6):109737. doi: 10.1016/j.isci.2024.109737 (PMC11126884; doi:10.1016/j.isci.2024.109737)
Supplement: Document S1. Figures S1–S8 [file mmc1.pdf]

iScience, Volume *27*

Supplemental information

STAT3 drives the expression

of ACSL4 in acute kidney injury

Virginie Poindessous, Helene Lazareth, Gilles Crambert, Lydie Cheval, Julio L. Sampaio, and Nicolas Pallet

FIGURE S1. [*Gpx4*, *Fsp1* and *Slc7a11* transcripts levels] related to FIGURE 1

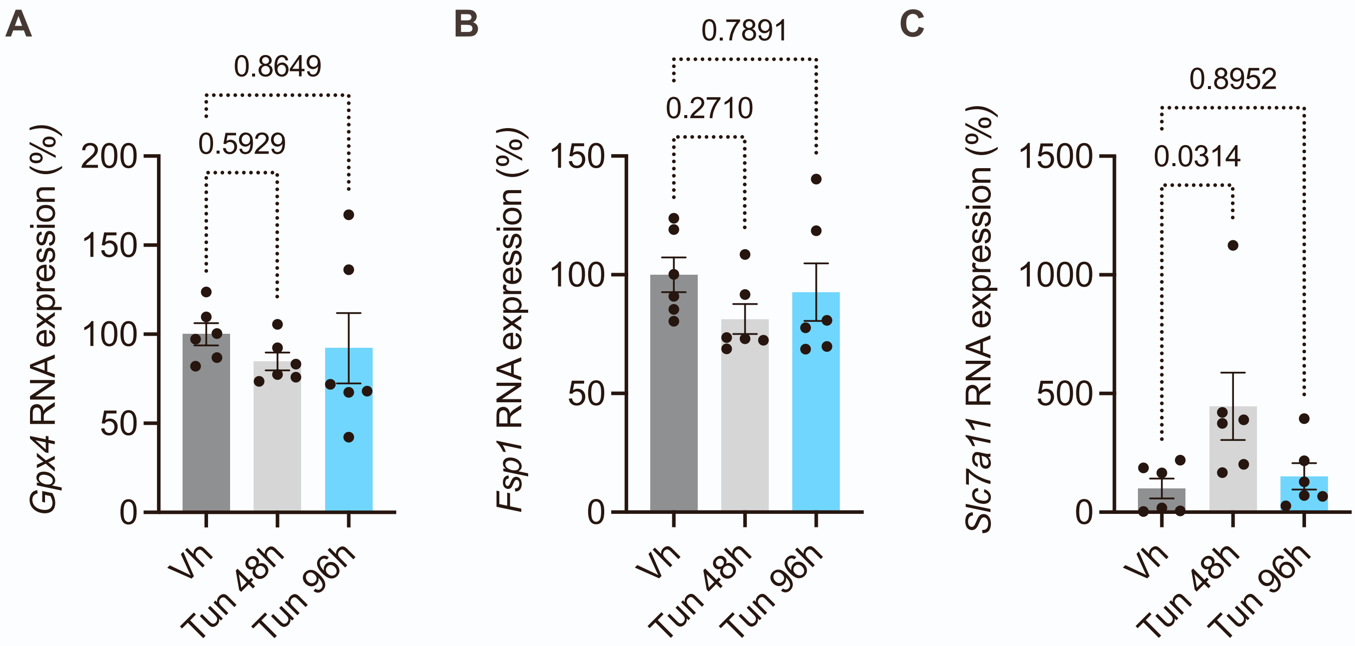

FIGURE S2. [STAT3 and CEBP binding sites] related to FIGURE 2

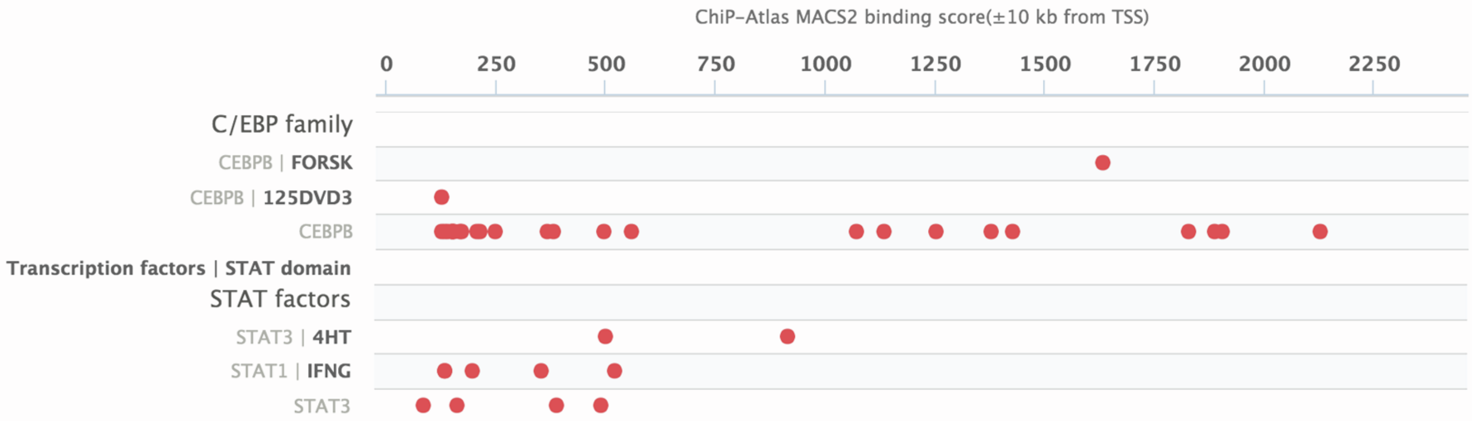

FIGURE S3. [IL-6 family receptors expression] related to FIGURE 3

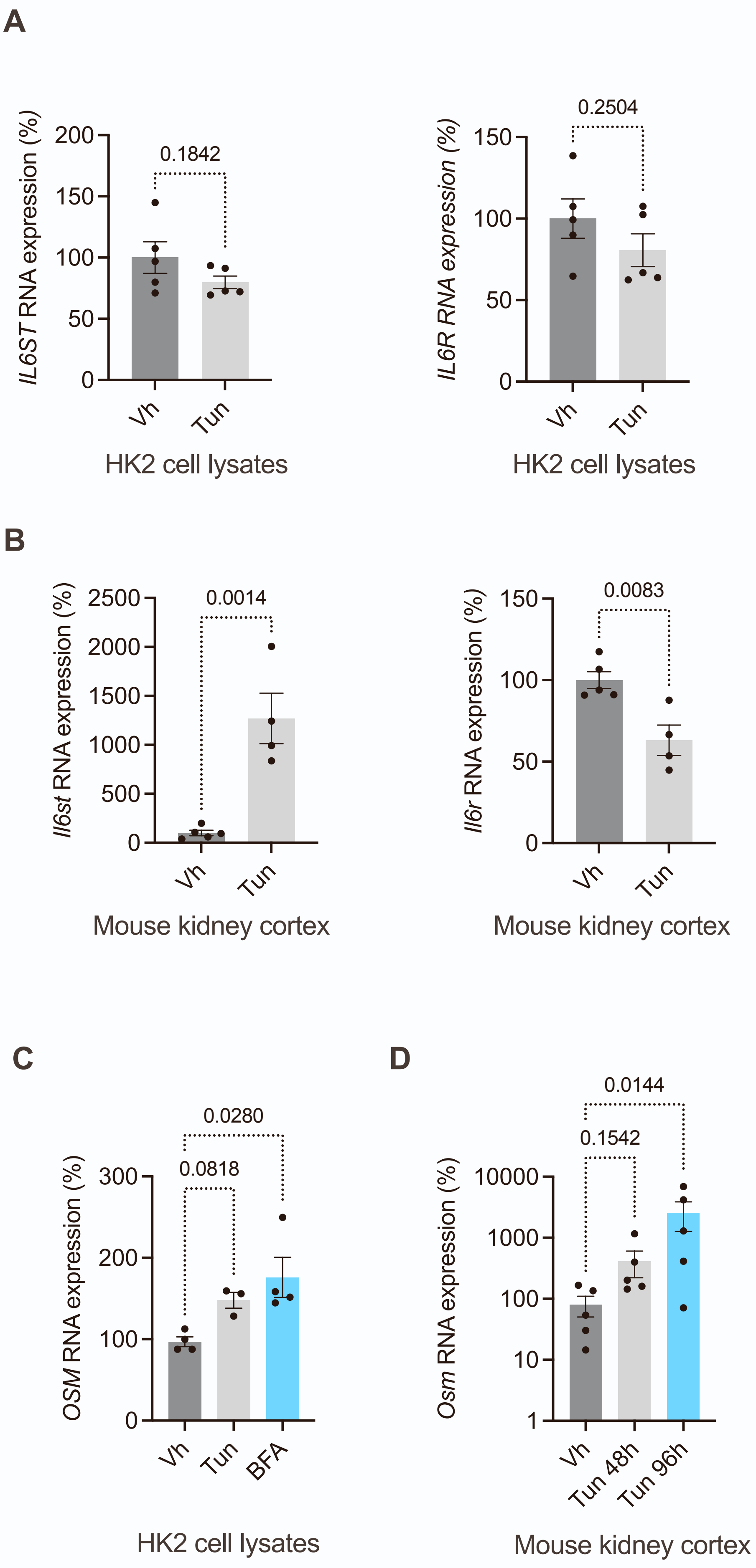

FIGURE S4. [ACSL4 and IDO1 expression by IFNg] related to FIGURE 4

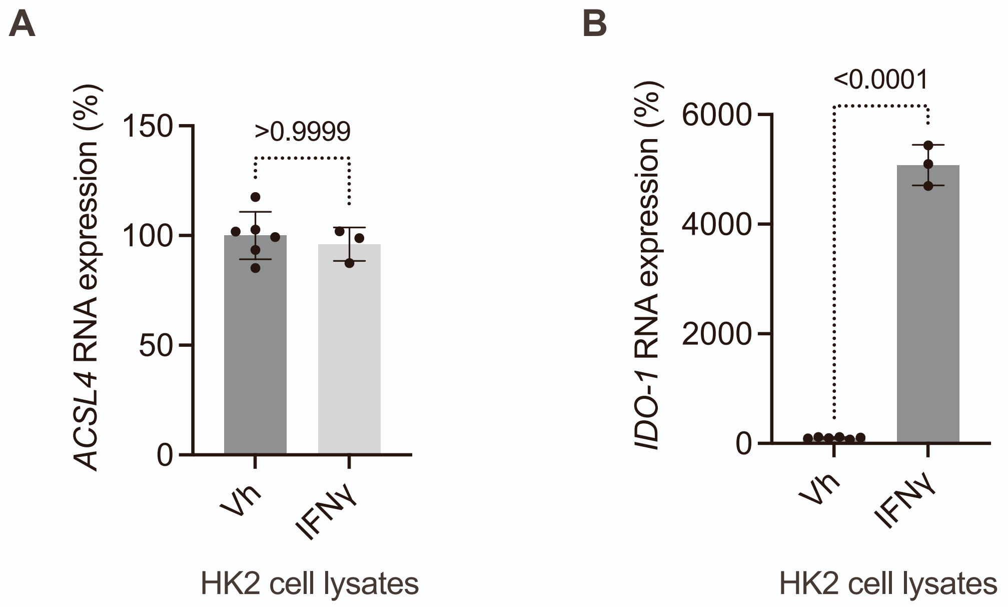

FIGURE S5. [Proximal tubule lipidome with tunicamycin] related to FIGURE 5

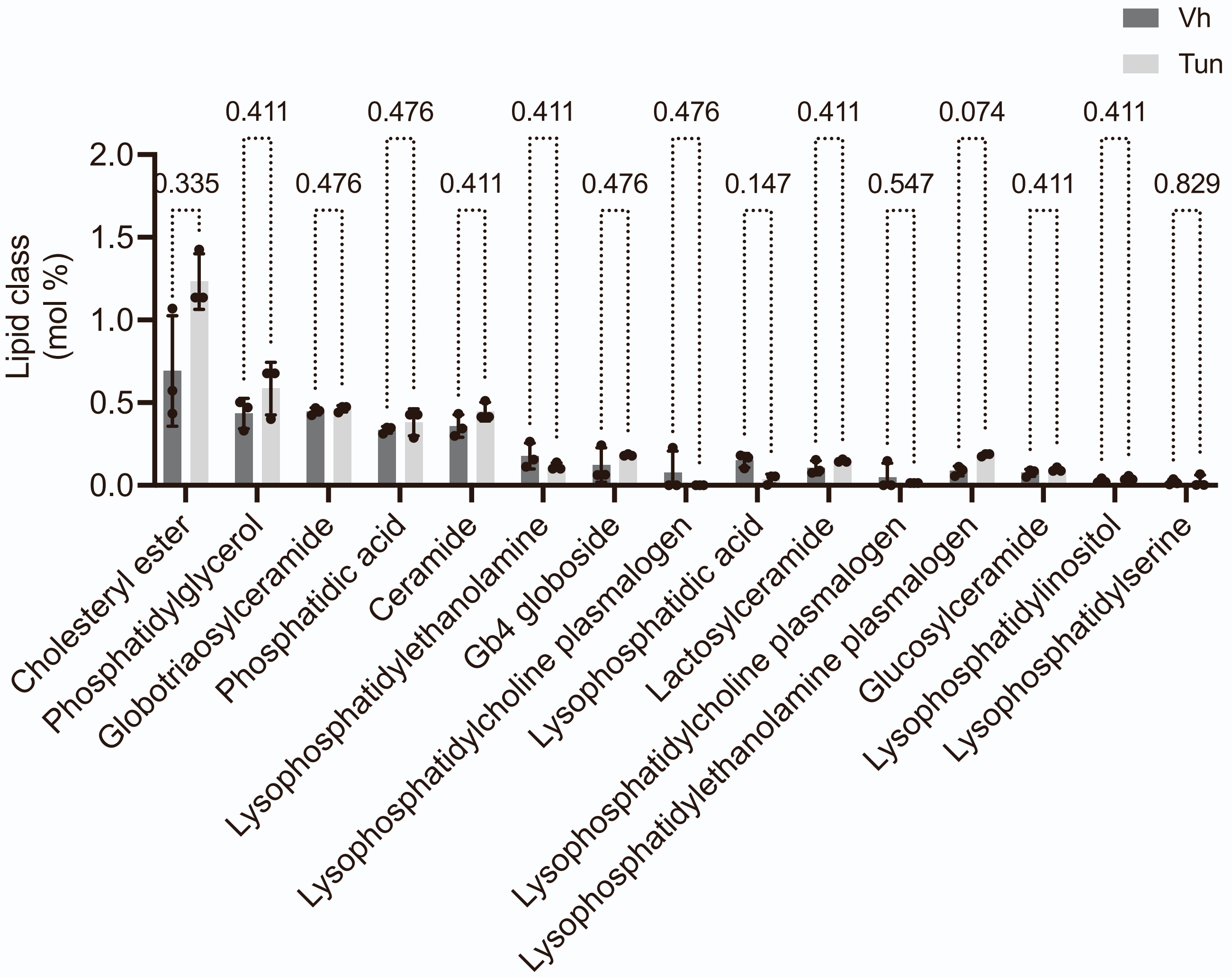

FIGURE S6. [Liperfluo staining of HK2 cells with RSL3] related to FIGURE 6

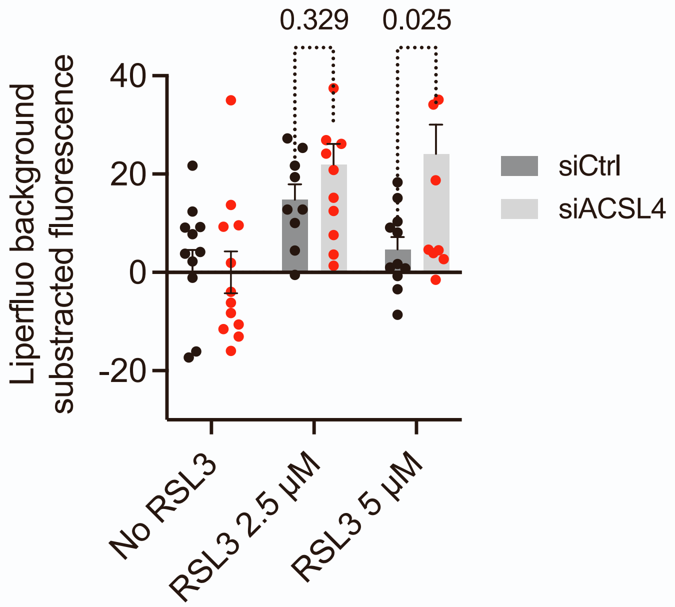

FIGURE S7. [HK2 cells lipidome with oncostatin] related to FIGURE 6

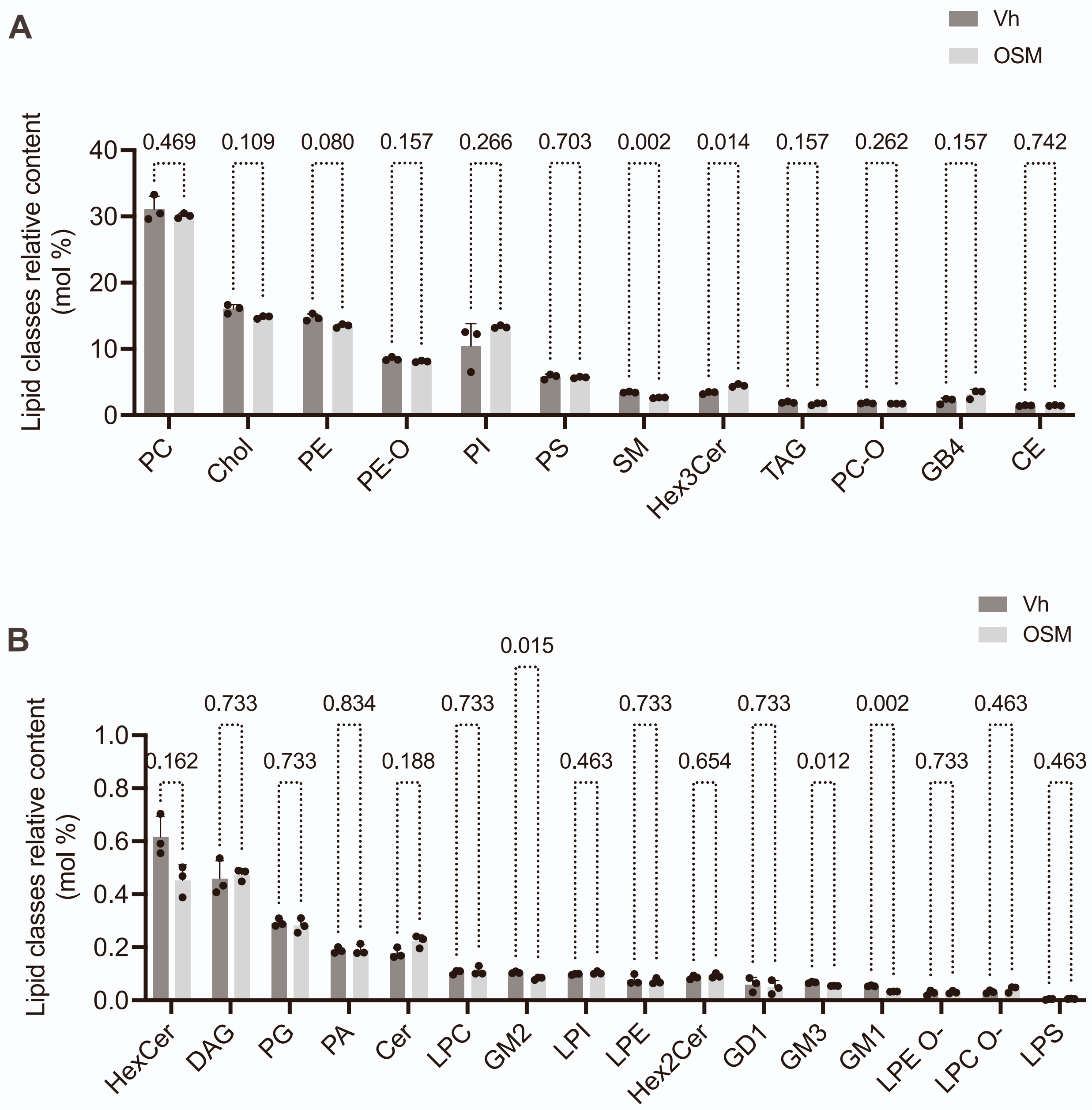

FIGURE S8. [HK2 cells lipidome with IL-6] related to FIGURE 6

A

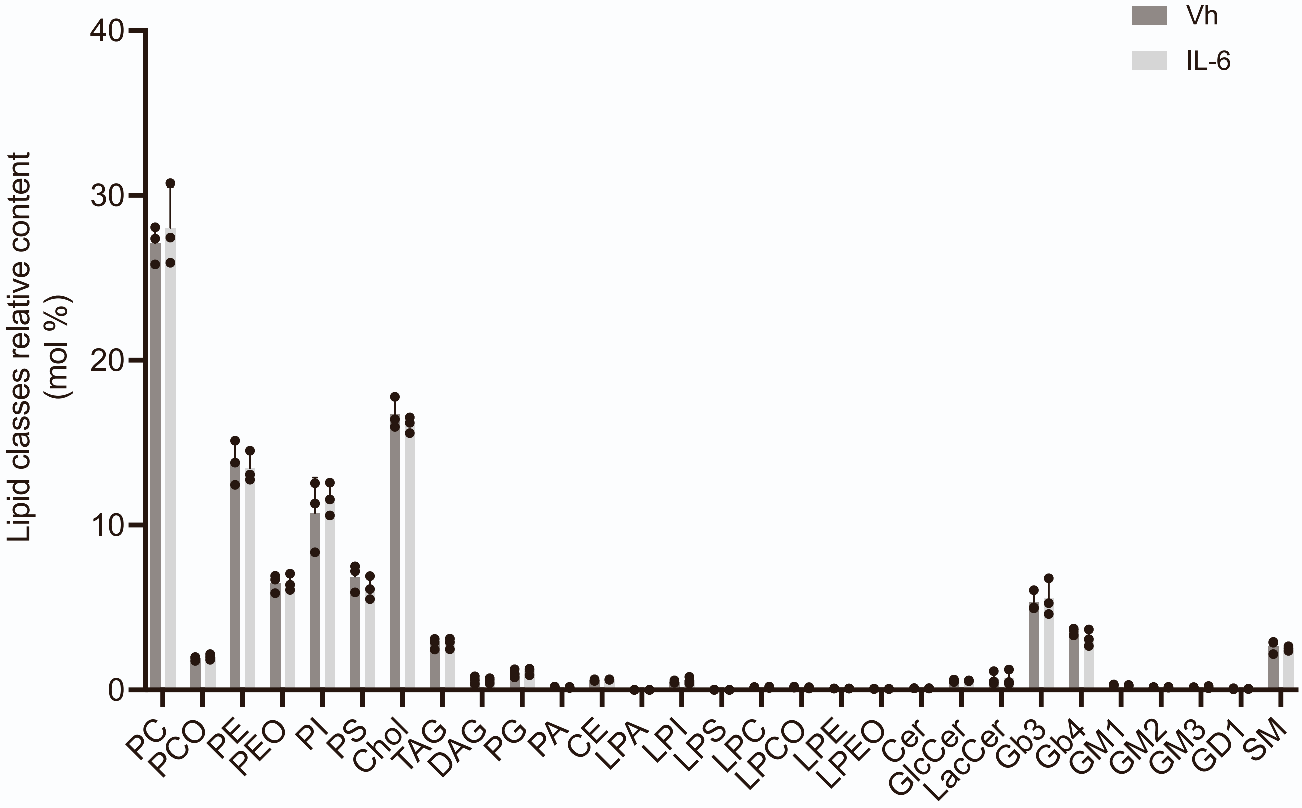

B

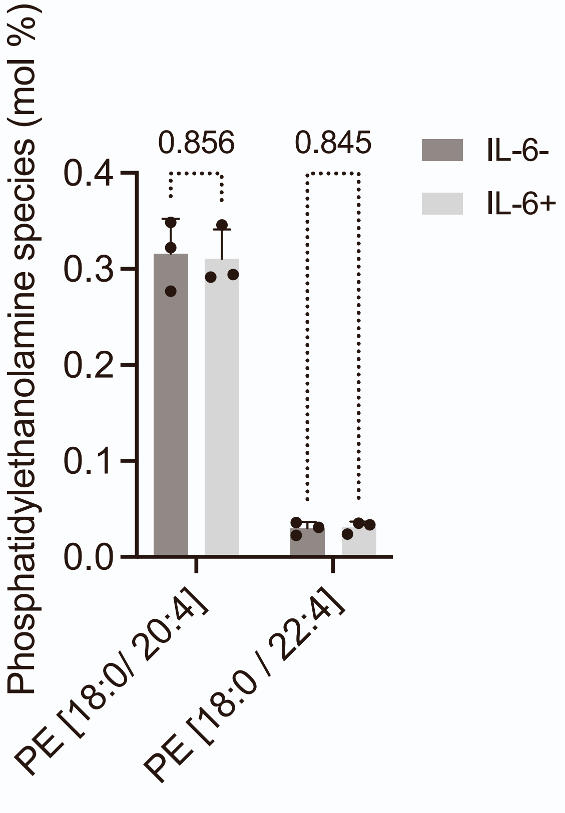

FIGURE S1. [*Gpx4*, *Fsp1* and *Slc7a11* transcripts levels] related to FIGURE 1
A, B and C. *Gpx4*, *Fsp1* and *Slc7a11* transcripts levels measured by q-PCR in kidneys cortex of mice 48 and 96 hours after intraperitoneal injection of 1 mg/kg tunicamycin (Tun) or DMSO (Vh) (n=6 mice per group). p values were computed with one-way ANOVA followed by a Dunnett's multiple comparisons test.

FIGURE S2. [STAT3 and CEBP binding sites] related to FIGURE 2
Schematic of the STAT3 and CEBP binding sites in the promoter of human ACSL4 gene generated using ChiP-Seq datasets integrated and analysed by the Signalling Pathways Project, a multi-omics knowledge mine based upon public, manually curated transcriptomic and cistromic (ChIP-Seq) datasets. http://www.signalingpathways.org/.

FIGURE S3. [IL-6 family receptors expression] related to FIGURE 3
A. *IL6ST (GP130)* and *IL6R* transcripts levels measured by q-PCR in HK-2 cells incubated with 100 ng/ml IL-6 for 24 hours (n=5 replicates per condition). p values were computed with a Student t test.
B. *Il6st (Gp130)* and *Il6r* transcripts levels measured by q-PCR in kidneys cortex of mice 48 hours after intraperitoneal injection of 1 mg/kg tunicamycin (Tun) or DMSO (Vh) (n=4-5 mice per group). p values were computed with a Student T test.
C. *OSM* transcripts levels measured by RT-qPCR in HK-2 cells incubated with 2.5 µg/ml tunicamycin (Tun), 5 µg/mL brefeldin A (BFA), or DMSO (Vh) for 24 hours (n=3 replicates per condition). p values were computed with one-way ANOVA followed by a Dunnett's multiple comparisons test.
D. Expression of *Osm* transcripts by q-PCR in kidneys cortex of mice 48 hours and 96 hours after intraperitoneal injection of 1 mg/kg Tunicamycin (Tun) or DMSO (Vh) (n=3 to 4 mice per group). p values were computed with one-way ANOVA followed by a Dunnett's multiple comparisons test.

FIGURE S4. [ACSL4 and IDO1 expression by IFNγ] related to FIGURE 4
A and B. *ACSL4* and *IDO1* transcripts levels measured by RT-qPCR in HK-2 cells incubated with 50 ng/ml IFNγ for 24 hours (n=4-5 replicates per condition). p values were computed with a Student's T test.

FIGURE S5. [Proximal tubule lipidome with tunicamycin] related to FIGURE 5
Relative contents in lipid classes with mol%<1 in isolated proximal tubules isolates from mouse kidney 48 hours after intraperitoneal injection of 1 mg/kg tunicamycin (Tun) or DMSO (Vh). (n=3 mice per group). p values were computed with the FDR approach with the method of Benjamini Krieger and Yekiuteli, with a FDR<1%. The y-axis indicates the relative expression of each lipid class (mol%), which is the percentage of total membrane lipids in the sample.

FIGURE S6. [Liperfluo staining of HK2 cells with RSL3] related to FIGURE 6
Fluorescence intensity of lipid hydroperoxides (LiperFluo staining) in HK-2 cells incubated with with RSL3 for 24 hours in the presence or absence of siRNA targeting *ACSL4* (siACSL4) or a scrambled siRNA (siCtrl) (n=12 replicates per condition). p values were computed with the FDR approach with the method of Benjamini, Krieger and Yekiuteli, with a FDR<1%.

FIGURE S7. [HK2 cells lipidome with oncostatin] related to FIGURE 6
A. Relative contents in lipid classes with mol%>1 in HK-2 cells incubated with 20 ng/ml OSM for 48 hours compared to the vehicle condition (n=3 replicates per condition). p values were computed with the FDR approach with the method of Benjamini, Krieger and Yekiuteli, with a FDR<1%. The y-axis indicates the relative expression of each lipid species (mol%), which is the percentage of total membrane lipids in the sample.
B. Relative contents in lipid classes with mol%<1 in HK-2 cells incubated with 20 ng/ml OSM for 48 hours compared to the vehicle condition (n=3 replicates per condition). p values were

computed with the FDR approach with the method of Benjamini, Krieger and Yekiuteli, with a FDR<1%. The y-axis indicates the relative expression of each lipid species (mol%), which is the percentage of total membrane lipids in the sample.

FIGURE S8. [HK2 cells lipidome with IL-6] related to FIGURE 6

A. Relative contents in lipid classes with mol%<1 in HK-2 cells incubated with 100 ng/ml Il-6 for 48 hours compared to the vehicle condition (n=3 replicates per condition). p values were computed with the FDR approach with the method of Benjamini Krieger and Yekiuteli, with a FDR<1%. The y-axis indicates the relative expression of each lipid class (mol%), which is the percentage of total membrane lipids in the sample.

B. Relative contents in PE (18:0 / 20:4) and PE (18:0 / 22:4) in HK-2 cells incubated with 100 ng/ml Il-6 for 48 hours compared to the vehicle condition (n=3 replicates per condition). p values were computed with the FDR approach with the method of Benjamini, Krieger and Yekiuteli, with a FDR<1%. PE: phosphatidylethanolamine, PC: phosphatidylcholine, PI: phosphatidylinositol. The y-axis indicates the relative expression of each lipid species (mol%), which is the percentage of total membrane lipids in the sample.
